# Neurokinin-1 receptor is an effective target for treating leukemia by inducing oxidative stress through mitochondrial calcium overload

**DOI:** 10.1073/pnas.1908998116

**Published:** 2019-09-05

**Authors:** Chentao Ge, Hemiao Huang, Feiyan Huang, Tianxin Yang, Tengfei Zhang, Hongzhang Wu, Hanwei Zhou, Qi Chen, Yue Shi, Yanfang Sun, Liangjue Liu, Xi Wang, Richard B. Pearson, Yihai Cao, Jian Kang, Caiyun Fu

**Affiliations:** ^a^Zhejiang Provincial Key Laboratory of Silkworm Bioreactor and Biomedicine, College of Life Sciences and Medicine, Zhejiang Sci-Tech University, 310018 Hangzhou, China;; ^b^Clinical Laboratory, Zhejiang Provincial Hospital of TCM, 310006 Hangzhou, China;; ^c^Department of Hematology, Zhejiang Province People’s Hospital, 310014 Hangzhou, China;; ^d^Department of Oncology, The People’s Liberation Army No. 903rd Hospital, 310013 Hangzhou, China;; ^e^Oncogenic Signalling and Growth Control Program, Peter MacCallum Cancer Centre, Melbourne, VIC 3000, Australia;; ^f^Sir Peter MacCallum Department of Oncology, University of Melbourne, Melbourne, VIC 3000, Australia;; ^g^Department of Microbiology, Tumor and Cell Biology, Karolinska Institute, 171 77 Stockholm, Sweden

**Keywords:** neurokinin-1 receptor, oxidative stress, mitochondrial calcium fluxes, leukemia

## Abstract

Substance P (SP) regulates multiple biological processes through its high-affinity neurokinin-1 receptor (NK-1R). While the SP/NK-1R signaling axis is involved in the pathogenesis of solid cancer, the role of this signaling pathway in hematological malignancy remains unknown. Here, we demonstrate that NK-1R expression is markedly elevated in the white blood cells from acute myeloid leukemia patients and a panel of human leukemia cell lines. Blocking NK-1R induces apoptosis in vitro and in vivo via increase of mitochondrial reactive oxygen species. This oxidative stress was triggered by rapid calcium flux from the endoplasmic reticulum into mitochondria and, consequently, impairment of mitochondrial function, a mechanism underlying the cytotoxicity of NK-1R antagonists. Besides anticancer activity, blocking NK-1R produces a potent antinociceptive effect in myeloid leukemia-induced bone pain by alleviating inflammation and inducing apoptosis. These findings thus raise the exciting possibility that the NK-1R antagonists, drugs currently used in the clinic for preventing chemotherapy-induced nausea and vomiting, may provide a therapeutic option for treating human myeloid leukemia.

## Significance

Despite tremendous efforts in developing effective therapeutics for treating acute myeloid leukemia (AML), this hematological disease remains an incurable malignancy. Here, we show surprising findings that neurokinin-1 receptor (NK-1R) is highly expressed in AML patients and that targeting NK-1R produced potent proapoptotic and antinociceptive effects. Given the clinical availability of the NK-1R antagonists for treating chemotherapy-induced adverse effects, the therapeutic effect of the NK-1R antagonists could be readily tested in human patients with myeloid leukemia. If the therapeutic effect is successfully validated in human patients, our findings would bring hope and benefits for millions of patients. Our study provides another example of drug discovery by mechanistic efforts.

Human myeloid leukemia, including acute myeloid leukemia (AML) and chronic myeloid leukemia (CML), is characterized by the expansion of abnormal white blood cells in the blood and bone marrow. The successful implementation of targeted therapies designed to inhibit the tyrosine kinase activity of the BCR-ABL oncoprotein has made a significant breakthrough in the treatment of CML patients. However, tyrosine kinase inhibitors (TKIs) do not cure CML, and CML remains a chronic disease with patients requiring TKI treatment for life (1). Some patients with CML progress to an accelerated phase with only a 7- to 11-mo median survival (2). In contrast, to date, there have been very few breakthroughs in the treatment of AML, particularly relapsed or refractory AML, and AML remains an incurable malignancy (3). Therefore, there is an urgent need for therapeutics to treat myeloid leukemia.

Substance P (SP) belongs to the tachykinin family of neuropeptides (4). It is widely distributed throughout the nervous and immune systems and regulates many pathophysiological processes (5). The biological actions of SP are mediated through binding to neurokinin receptors, members of the G protein-coupled receptor (GPCR) family that includes neurokinin-1 receptor (NK-1R), NK-2R, and NK-3R. NK-1R confers the highest affinity binding for SP (5). Dysregulation of the SP/NK-1R system contributes to multiple pathological processes, including pain, chronic inflammation, affective and addictive disorders, and cancer (6, 7). It is now becoming clear that SP/NK-1R signaling plays an important role in cancer pathogenesis. The SP/NK-1R system is considered as an independent therapeutic target for cancer treatment (8). Elevated expression of SP/NK-1R has been identified in multiple cancer types and promotes angiogenesis, proliferation, and metastasis of solid tumor cells, including breast (9–13), gastric (14, 15), liver (16–18), colon (19), and pancreatic (20–22) cancer, as well as melanoma (23), in an autocrine, paracrine, or neurocrine manner. More importantly, one of the NK-1R antagonists, Aprepitant, has been approved by the US Food and Drug Administration for the treatment of nausea and vomiting caused by cancer chemotherapy (24, 25). SR140333 is another highly selective and potent NK-1R antagonist with a different chemical structure from Aprepitant by featuring a piperidine scaffold, and its analog, SSR-240600, is in a phase II clinical trial for the treatment of overactive bladder syndrome (26, 27). Nevertheless, the role of NK-1R in the pathogenesis of human hematological malignancy has not been systematically characterized, although one study showed inhibition of cell proliferation by NK-1R antagonists in acute lymphoblastic leukemia (28). Moreover, the molecular mechanisms underlying the antitumor action of NK-1R antagonists remain elusive. By analyzing NK-1R gene expression in Gene Expression Profiling Interactive Analysis (http://gepia.cancer-pku.cn/), a webserver for gene expression profiling based on The Cancer Genome Atlas data (29), we found up-regulated NK-1R messenger RNA (mRNA) expression in AML patients compared with normal controls, implicating a potential pathological role of NK-1R in AML. In addition, the important role of SP/NK-1R in nociceptive transmission has been extensively researched for decades. It is well known that SP is released from the primary sensory neurons of peripheral nerves and the spinal cord and that it induces pain transmission upon binding to the high-affinity NK-1R (5). NK-1R antagonists have shown an antinociceptive effect in inflammation or nerve damage-induced pain mouse models despite lack of efficacy in producing analgesia in clinical trials (30). As bone pain is a common symptom in cancer patients (31) and occurs in leukemia patients when the bone marrow expands due to the accumulation of abnormal white blood cells (32), it will be interesting to explore whether NK-1R antagonists can simultaneously abrogate this clinical symptom of leukemia patients.

We herein demonstrated that NK-1R protein expression level was elevated in AML patients, blocking NK-1R function-induced apoptosis of myeloid leukemia cells and inhibiting tumor growth by inducing oxidative stress via mitochondrial calcium overload. Meanwhile, NK-1R inhibition alleviated CML-induced bone pain in a mouse model that was associated with its antiinflammation and proapoptosis activities. These findings therefore provide a strong rationale for repurposing well-tolerated NK-1R antagonists for human myeloid leukemia treatment.

## Results

### The NK-1R Protein Expression Level Is Elevated in Human Myeloid Leukemia Patients and Cell Lines.

**Fig. 1.**
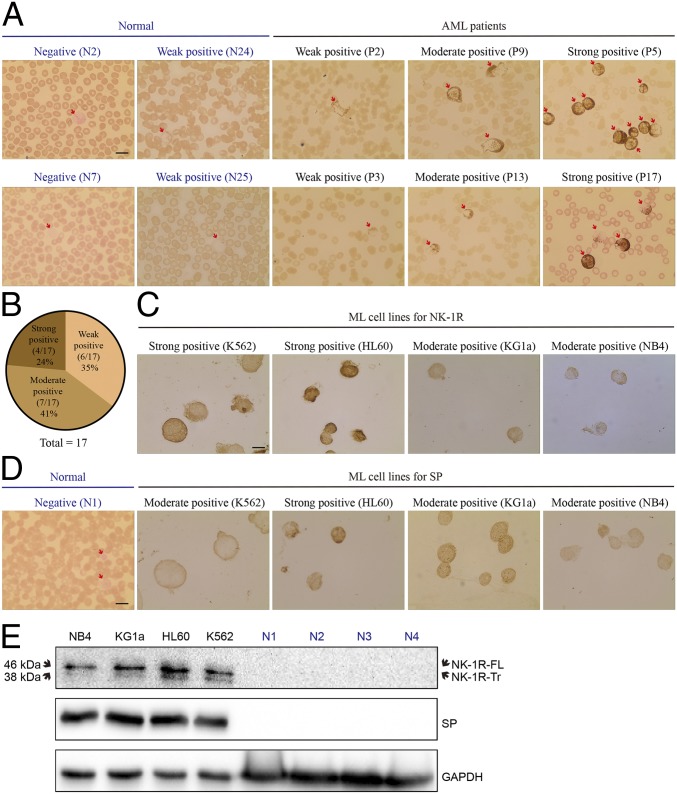
NK-1R protein expression level is elevated in human AML patients and cell lines. (*A*) Representative images of immunocytochemical analysis of NK-1R in peripheral blood samples of 17 AML patients (P) and 25 normal healthy volunteers (N). The red arrows indicate white blood cells. (Magnification: 1,000×.) The intensity of positively stained cells was scored using a scale of 0 (negative), 1 (weak), 2 (moderate), and 3 (strong). (Scale bar: 20 μM.) All images of 17 AML patients and 25 normal healthy volunteers are shown in *SI Appendix*, Fig. S1*A*, and the patient information is listed in *SI Appendix*, Table S1. (*B*) Pie chart overview of NK-1R positivity in all investigated AML samples (*n* = 17). (*C*) Representative images of immunocytochemical analysis of NK-1R in myeloid leukemia (ML) cell lines. (Scale bar: 20 μM.) (*D*) Representative images of immunocytochemical analysis of SP in myeloid leukemia cell lines, as well as in 1 normal healthy volunteer. All images of 10 normal healthy volunteers are shown in *SI Appendix*, Fig. S1*B*. (Scale bar: 20 μM.) (*E*) Western blotting of NK-1R, including a full-length isoform (NK-1R-FL) and a truncated isoform (NK-1R-Tr), and SP in the white blood cells of normal healthy volunteers and human myeloid leukemia cell lines.

To explore its functional role in hematological malignancies, we first assessed NK-1R expression in the peripheral blood samples of 17 patients with newly diagnosed AML (*SI Appendix*, Table S1) and 25 healthy volunteers by immunocytochemistry (Fig. 1*A* and *SI Appendix*, Fig. S1*A*). All AML patients showed positive expression of NK-1R, including 35% of AML patients with weak expression, 41% with moderate expression, and 24% with strong expression (Fig. 1*B*). In contrast, only 8% of control samples (2 of 25 normal healthy samples) showed weak expression of NK-1R (Fig. 1*A* and *SI Appendix*, Fig. S1*A*). NK-1R expression was confined to the peripheral white blood cells and mostly located on the cell membrane and cytoplasm (Fig. 1*A* and *SI Appendix*, Fig. S1*A*).

We also examined the protein expression levels of SP and NK-1R in 3 human AML cell lines (NB4, KG-1α, and HL60) and one CML cell line (K562) by immunocytochemistry. All myeloid leukemia cell lines presented moderate to strong expression of NK-1R (Fig. 1*C*) and SP (Fig. 1*D* and *SI Appendix*, Fig. S1*B*), whereas lack of positive staining for both NK-1R and SP was observed in all 10 healthy volunteers. The protein expression levels of SP and NK-1R in myeloid leukemia cell lines were further confirmed by Western blotting. Two isoforms of NK-1R have been identified and characterized, including a full-length receptor and a truncated receptor with molecular weights of 46 kDa and 38 kDa, respectively (33). Consistently, all cell lines expressed higher levels of NK-1R and SP than healthy volunteers (Fig. 1*E*), supporting a potential functional role for the NK-1R/SP system in the progression of human myeloid leukemia.

### Blocking NK-1R Induces Apoptosis in Human Myeloid Leukemia Cells.

**Fig. 2.**
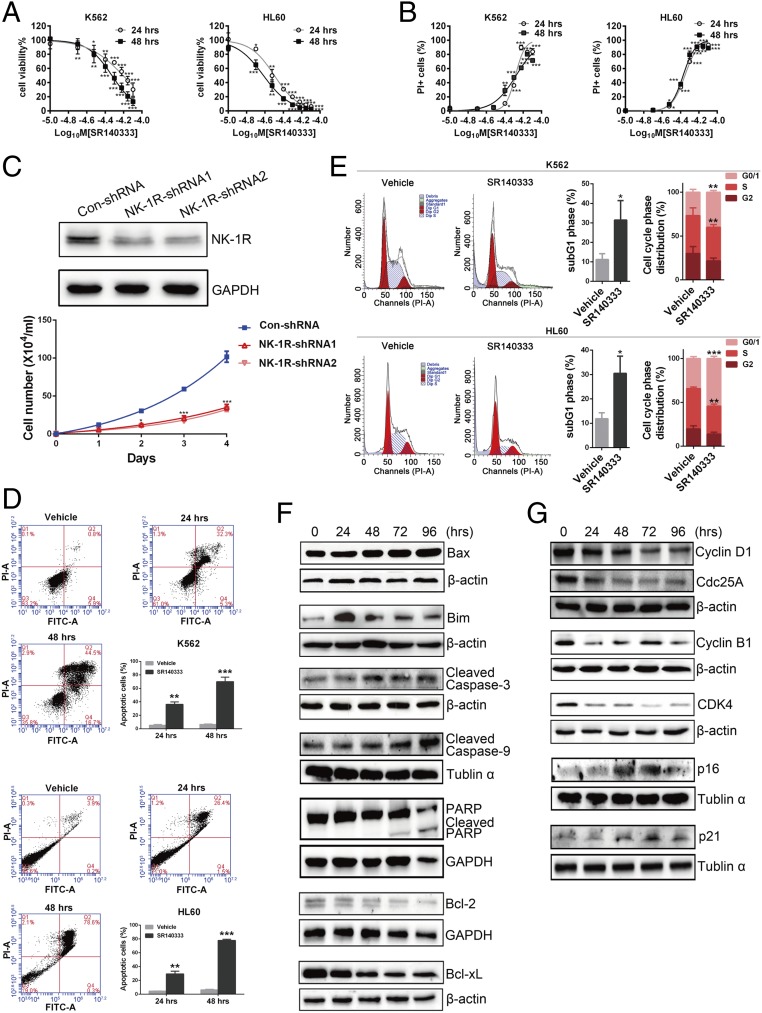
Blocking NK-1R induces apoptosis in human myeloid leukemia cells. (*A*) Cell viability after treatment with SR140333 at the indicated doses for 24 h and 48 h. The cell viability was calculated as the percentage of live cells in the drug treatment group relative to the vehicle-treated group. The live cells were counted by Trypan blue exclusion. Values represent mean ± SEM (*n* = 3). *P < 0.05, **P < 0.01, ***P < 0.001 (compared with the vehicle-treated cells). (*B*) Percentage of PI-positive cells after treatment with SR140333 at the indicated doses for 24 h and 48 h. Values represent mean ± SEM (*n* = 3). *P < 0.05, **P < 0.01, ***P < 0.001 (compared with the vehicle-treated cells). (*C*) NK-1R expression levels and cell viability after short hairpin RNA (shRNA)-mediated depletion of NK-1R in K562 cells. Values represent mean ± SEM (*n* = 3). *P < 0.05, ***P < 0.001 (compared with the Con-shRNA group). Con, control. (*D*) Annexin-V plus PI analysis of K562 and HL60 cells treated with SR140333 at 33 μM and 18 μM, respectively. The percentage of apoptotic cells was the proportion of early apoptotic cells (Q4 area) and later apoptotic cells (Q2 area). Values represent mean ± SEM (*n* = 3). **P < 0.01, ***P < 0.001 (compared with the vehicle-treated group). FITC, fluorescein isothiocyanate. (*E*) Cell cycle analysis of K562 and HL60 cells treated with SR140333 at 33 μM and 18 μM, respectively, for 24 h. Values represent mean ± SEM (*n* = 3). *P < 0.05; **P < 0.01, ***P < 0.001 (compared with the vehicle-treated group). Western blotting of apoptosis-related (*F*) and cell cycle-related (*G*) proteins in K562 cells treated with SR140333 at 33 μM is shown. GAPDH, Tubulin α, and β-actin were used as the loading controls. For the proteins probed in the same membrane (Cyclin D1 and Cdc25A), 1 loading control was used. The protein quantification is shown in *SI Appendix*, Fig. S3.

The up-regulation of NK-1R expression in AML patients led us to perform an in-depth characterization of NK-1R function in the CML cell line K562 and the AML cell line HL60. Treatment with the NK-1R antagonist SR140333 resulted in a dose-dependent inhibition of cell proliferation (Fig. 2*A*) and induction of cell death (Fig. 2*B*) in both the K562 and HL60 cell lines. The cytotoxic effect was also observed following treatment with Aprepitant (*SI Appendix*, Fig. S2 *A* and *B*). Consistent with this data, depletion of NK-1R by short hairpin RNA in K562 cells significantly reduced cell viability (Fig. 2*C*). Cell death in response to NK-1R inhibition was concomitant with induction of apoptotic markers, including increased Annexin-V/propidium iodide (PI) staining (Fig. 2*D*), and increased the percentage of cells with sub-G_1_ content (Fig. 2*E*)

Consistent with the cytotoxicity of SR140333, treatment of K562 with SR140333 resulted in increased expression of the proapoptotic proteins Bax and Bim, cleaved Caspase-3 and cleaved Caspase-9, as well as cleaved PARP, and a decrease in the abundance of antiapoptotic proteins Bcl-2 and Bcl-xL (Fig. 2*F* and *SI Appendix*, Fig. S3*A*), indicating that blocking NK-1R activates the intrinsic apoptotic pathways.

We also observed a cell cycle arrest in SR140333-treated K562 and HL60 cells, as evidenced by a significant increase of cell population in G_0_/G_1_ phase and a significant decrease of cells in S phase after 24 h of treatment (Fig. 2*E*). Consistent with this observation, the expression levels of Cyclin D1, Cyclin B1, CDK4, and CDC25A were decreased significantly, whereas the CDK inhibitors p16 and p21 were increased in response to SR140333 treatment (Fig. 2*G* and *SI Appendix*, Fig. S3*B*).

### Blocking NK-1R Inhibits Human Myeloid Leukemia Xenograft Growth.

**Fig. 3.**
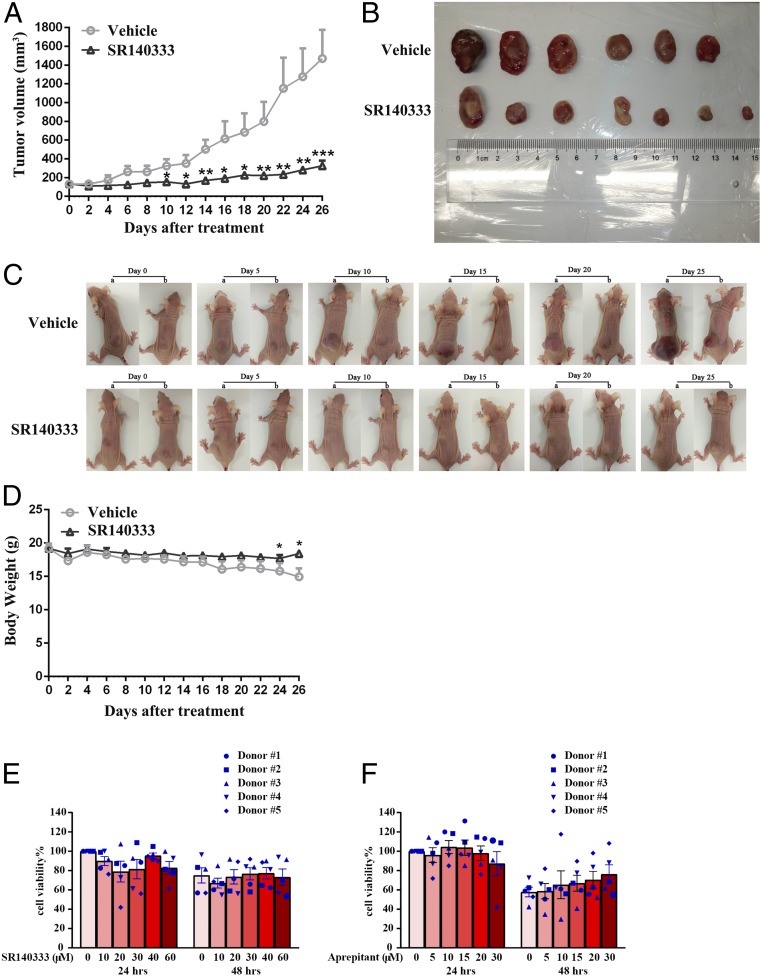
Blocking NK-1R inhibits human myeloid leukemia xenograft growth in vivo. Female BALB/c nude mice implanted with K562 cells were treated with SR140333 as described in *Materials and Methods*. (*A*) Tumor volume measured in K562 xenografts. Values represent mean ± SEM (*n* = 6 for vehicle group and *n* = 7 for SR140333 group). *P <0.05, **P <0.01, ***P <0.001 (compared with the vehicle-treated group). (*B*) Image of tumors excised from all mice on day 26 (*n* = 6 mice for the vehicle-treated group and *n* = 7 mice for SR140333 treatment group). (*C*) Images of the representative mice bearing K562 tumors in the vehicle-treated group and SR140333 treatment group throughout the entire experiment. (*D*) Body weight measured in K562 tumor-bearing mice. Values represent mean ± SEM (*n* = 6 for vehicle-treated group and *n* = 7 for SR140333 group). *P < 0.05 (compared with the vehicle-treated group). Cell viability after treatment with SR140333 (*E*) or Aprepitant (*F*) is shown at the indicated doses for 24 h and 48 h on human normal CD34^+^ hematopoietic cells.

The in vivo effect of blocking NK-1R on human myeloid leukemia cell growth was examined in the K562 xenograft mouse model. A daily regimen of SR140333 treatment (10 mg/kg) via in situ injection dramatically reduced the tumor volume (Fig. 3 *A*–*C*). In addition, there were no serious side effects after administration of SR140333, with no difference in weight gain between drug treatment and vehicle groups until day 24 (Fig. 3*D*) and lack of detectable growth-inhibitory effect in normal mouse bone marrow cells (*SI Appendix*, Fig. S4*A*).

Furthermore, there was no proliferation-inhibitory effect observed in human normal CD34^+^ hematopoietic cells from 5 independent donors in the presence of SR140333 (Fig. 3*E*) or Aprepitant (Fig. 3*F*) and no hemolytic toxicity in human red blood cells (*SI Appendix*, Fig. S4*B*), indicating selective targeting of tumor cells by NK-1R antagonists. Our results thus provided preclinical evidence to support the efficacy and safety of NK-1R antagonists as anticancer drugs.

### Blocking NK-1R Induces Mitochondrial Oxidative Stress.

**Fig. 4.**
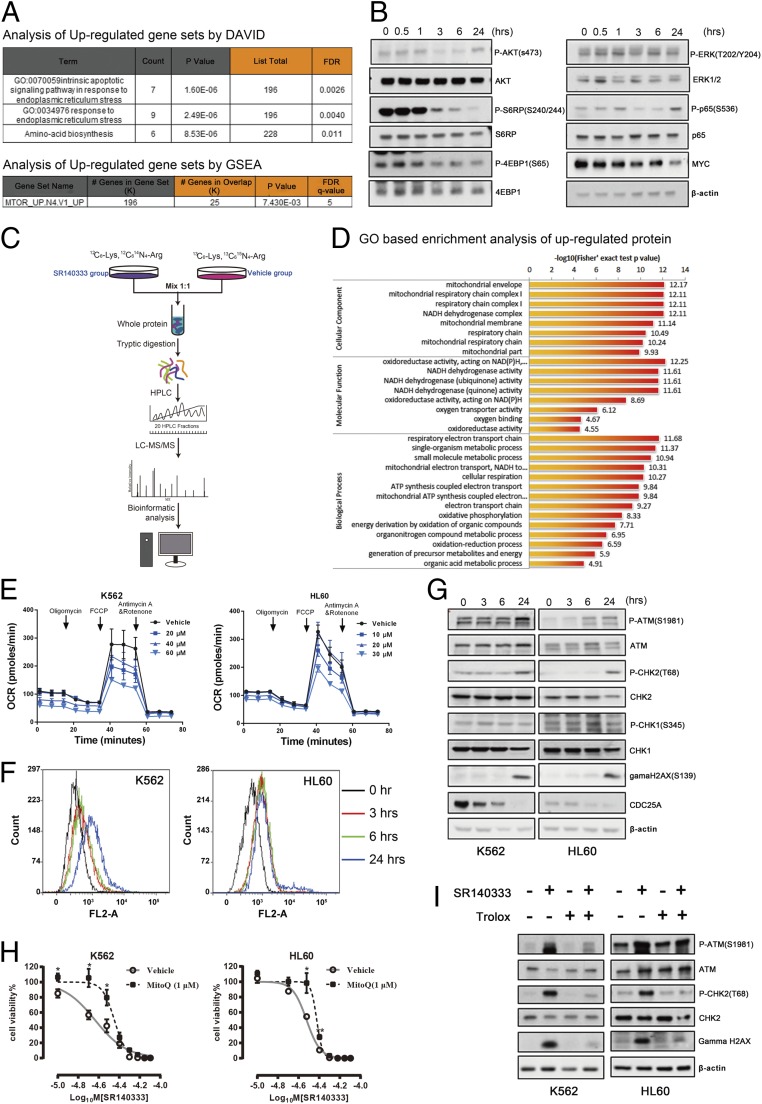
Blocking NK-1R induces mitochondrial oxidative stress. (*A*) GO analysis of RNA sequencing from K562 cells treated or untreated with SR140333 at 33 μM for 12 h. DAVID, Database for Annotation, Visualization, and Integrated Discovery; FDR, false discovery rate; GSEA, gene set enrichment analysis. (*B*) Western blotting of the proteins involved in AKT-mTORC1, ERK, NF-kappa B, and MYC pathways in K562 cells treated with SR140333 at 40 μM at the indicated time points. β-Actin was used as the loading control. (*C*) Schematic illustration of the experimental design of SILAC quantitative proteomic profiling. LC-MS/MS, liquid chromatography-tandem MS. (*D*) GO analysis showing the most significant GO terms. NAD(P)H, nicotinamide adenine dinucleotide phosphate. (*E*) OCR determination using a Seahorse XF96 Extracellular Flux analyzer in K562 and HL60 cells treated with SR140333 at the indicated concentrations for 3 h. Values are mean ± SEM (*n* = 3). (*F*) Mitochondrial superoxide levels in K562 and HL60 cells treated with SR140333 at 60 μM and 30 μM, respectively, measured by MitoSOX Red. (*G*) Western blotting of DDR-associated proteins in K562 and HL60 cells treated with SR140333 at 60 μM and 30 μM, respectively, for the indicated time periods. β-Actin was used as the loading control. (*H*) K562 and HL60 cells were pretreated with MitoQ (1 μM) for 1 h, and then treated with SR140333 at the indicated doses for 24 h. The cell viability was calculated as the percentage of live cells in the drug treatment group relative to the vehicle-treated group. The live cells were counted by Trypan blue exclusion. Values are mean ± SEM (*n* = 3). *P < 0.05 (compared with the group treated with SR140333 alone). (*I*) Western blotting of DDR-associated proteins in K562 and HL60 cells pretreated with Trolox at 100 μM and then treated with SR140333 at 60 μM and 30 μM, respectively, for 24 h. β-Actin was used as the loading control.

To investigate the molecular mechanisms of the proapoptotic effect of NK-1R antagonism in myeloid leukemia, we performed RNA sequencing to analyze the global mRNA transcriptome signature in K562 cells 12 h after SR140333 treatment. A total of 381 transcripts were differentially expressed, in which 294 genes were significantly up-regulated and 87 genes were significantly down-regulated (false discovery rate less than 0.05 and log_2_-fold change ratio more than 1; Dataset S1). The gene ontology (GO) analysis determined by the Database for Annotation, Visualization, and Integrated Discovery revealed a significant enrichment in an intrinsic apoptotic signaling pathway and the response to endoplasmic reticulum (ER) stress (Fig. 4*A*). We also performed a standard gene set enrichment analysis with the Molecular Signature Database. The analysis revealed the most significant pathway enriched in the SR140333-treated cells was mammalian target of rapamycin (mTOR) signaling (Fig. 4*A*). Indeed, at 3 h posttreatment, a rapid decrease of S6RP phosphorylation (S240/244) and 4EBP1 phosphorylation (S65), downstream molecules of the mTOR complex 1 (mTORC1), was detected (Fig. 4*B*). It is noted that SR140333 also acutely suppressed MYC expression (Fig. 4*B*). Induction of nuclear factor (NF)-kappa B and extracellular signal-regulated kinase (ERK) signaling pathways has been reported upon NK-1R activation (34–36). Interestingly, the phosphorylation level of p65, a component of the NF-kappa B pathway, was increased at 24 h posttreatment and ERK phosphorylation was not changed. Therefore, it is unlikely that these 2 pathways participate in SR140333-induced cell death. Interestingly, the mTORC1 inhibitor Everolimus showed only a very mild inhibitory effect on cell proliferation and did not induce cell death (*SI Appendix*, Fig. S5), indicating mTORC1 signaling is not a key mediator of SR140333-induced cytotoxicity.

We further performed stable isotope labeling by/with amino acids in cell culture (SILAC)-mass spectrometry (MS)–based quantitative proteomics analysis to explore the molecular responses induced by SR140333 (Fig. 4*C*). There are 713 proteins significantly up-regulated and 401 proteins significantly down-regulated (a ratio >1.3 is considered as up-regulation and a ratio <0.77 is considered as down-regulation; Dataset S2) after 24 h of treatment. The GO-based enrichment analysis of up-regulated proteins revealed an increase of electron transport chain and oxidative phosphorylation (Fig. 4*D* and *SI Appendix*, Fig. S6*A*), suggesting NK-1R inhibition may affect mitochondrial function. The increase of reduced nicotinamide-adenine dinucleotide (NADH)/Ubiquinone Oxidoreductase Subunit A8 (NDUFA8) and NADH/Ubiquinone Oxidoreductase Subunit B8 (NDUFB8) in complex I and cytochrome *c* oxidase copper chaperone (COX17) in complex IV following SR140333 treatment was validated by Western blotting (*SI Appendix*, Fig. S6*B*).

To further determine the effect of NK-1R inhibition on mitochondrial function, we examined the oxidative phosphorylation status by measuring the oxygen consumption rate (OCR) 3 h after treatment with SR140333. At this time point, mitochondrial membrane permeability was unaffected (*SI Appendix*, Fig. S7*A*), suggesting cells still maintained mitochondrial integrity. Interestingly, a rapid decrease of baseline OCR, adenosine 5′-triphosphate (ATP) production, and proton leak were observed in a dose-dependent manner (Fig. 4*E* and *SI Appendix*, Fig. S7*B*), suggesting that the tumor-suppressive effect of SR140333 may be associated with impaired mitochondrial function and energy production.

As mitochondria are the major intracellular sources of reactive oxygen species (ROS) production and decreased mitochondrial function may disrupt mitochondrial redox homeostasis (37), we measured the mitochondrial superoxide production by flow cytometric analysis using MitoSOX, a redox-sensitive fluorogenic probe specifically targeting mitochondria. Treatment with SR140333 (Fig. 4*F*) or Aprepitant (*SI Appendix*, Fig. S7*C*) resulted in an increase of the abundance of mitochondrial oxidants as early as 3 h posttreatment. Interestingly, Linley et al. (38) reported that NK-1R activation increased mitochondrial ROS production. However, we did not observe a significant stimulatory effect on ROS production by SP in K562 and HL60 cells (*SI Appendix*, Fig. S7*D*).

ROS accumulation causes damage to DNA, proteins, and lipids, and thus inhibits cell proliferation and induces cell death (39). This oxidative stress can induce activation of the DNA damage response (DDR) pathways including ATM-CHK2 and ATR-CHK1 checkpoints (40). ATM phosphorylates multiple substrates, including CHK2 on threonine 68 and the histone variant H2AX on serine 139 (gamma H2AX). CHK2 phosphorylates multiple substrates involved in cell cycle progression, including CDC25A, where its phosphorylation leads to degradation through the ubiquitin-proteasome pathway, resulting in cell cycle arrest at G_1_-S phase. Alternatively, activation of ATR in response to DNA damage results in phosphorylation of CHK1 at serine residue 345. Our data indicate that blocking NK-1R with SR140333 increased the phosphorylation of ATM, CHK2, and H2AX and was accompanied by decreased levels of CDC25A (Fig. 4*G*), all consistent with activation of DDR-driven ATM-CHK2 signaling by oxidative stress contributing to the antitumor effects of NK-1R antagonists.

To further confirm that ROS is involved in SR140333-induced cell death, we used the ROS scavenger Trolox and the mitochondrial-targeted antioxidant MitoQ. The Trolox (*SI Appendix*, Fig. S7*E*) and MitoQ (Fig. 4*H*) efficiently increased cell survival and Trolox blocked phosphorylation of ATM and CHK2, as well as H2AX (Fig. 4*I*). Similarly, Trolox increased cell viability in the presence of another NK-1R antagonist, Aprepitant (*SI Appendix*, Fig. S7*E*). These results thus firmly support that ROS production mediates the cytotoxicity of NK-1R inhibitors.

### ER-Mitochondrial Calcium Overload Contributes to Oxidative Stress and Cell Apoptosis in Response to NK-1R Inhibition.

**Fig. 5.**
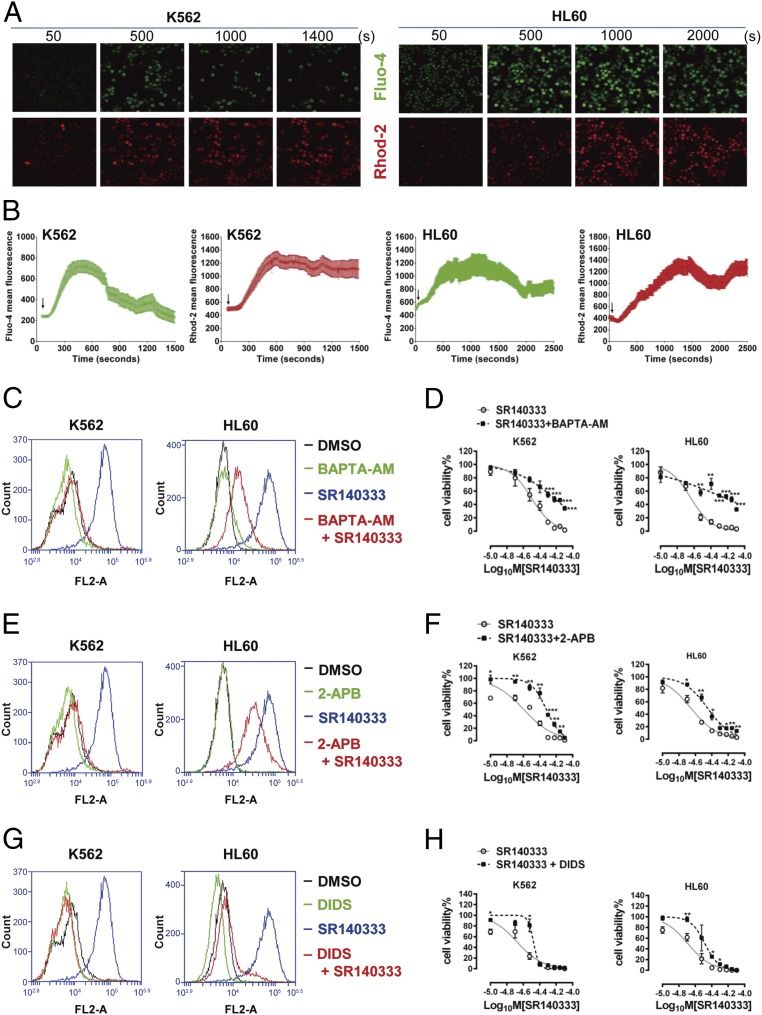
Mitochondrial calcium overload contributes to oxidative stress and cell death in response to NK-1R inhibition. (*A*) Images of cytosolic calcium indicator Fluo-4 AM staining and mitochondrial calcium indicator Rhod-2 AM staining in K562 and HL60 cells. After the initial measurement for 50 s to determine the baseline fluorescence, SR140333 at 60 μM for K562 cells and 30 μM for HL60 was added into the culture medium. Image acquisition continued for 1,500 to 2,500 s after drug treatment. (Magnification: 200×.) (*B*) Quantitative results of Fluo-4 AM and Rhod-2 AM fluorescence intensity in K562 and HL60 cells. Calcium concentrations were expressed as the average fluorescence intensity of 20 cells per field randomly from at least 3 fields at each time point. The arrow indicates the time to add SR140333. (*C* and *D*) K562 and HL60 cells were pretreated with BAPTA-AM (1 μM for K562 and 3 μM for HL60) for 1 h, and then treated with SR140333 at 60 μM and 30 μM, respectively. (*C*) Mitochondrial superoxide level was measured by MitoSOX 3 h after treatment. (*D*) Cell viability after treatment for 24 h. Values are mean ± SEM (*n* = 3). (*E* and *F*) K562 and HL60 cells were pretreated with 2-APB (1 μM for K562 and 6 μM for HL60) for 1 h, and then treated with SR140333 at 60 μM and 30 μM, respectively. The mitochondrial superoxide level (*E*) and the cell viability (*F*) are shown. Values are mean ± SEM (*n* = 3). (*G* and *H*) K562 and HL60 cells were pretreated with DIDS (2 μM for K562 and 6 μM for HL60) for 1 h, and then treated with SR140333 at 60 μM and 30 μM, respectively. The mitochondrial superoxide level (*G*) and the cell viability (*H*) are shown. Values are mean ± SEM (*n* = 3). (*D*, *F*, and *H*) *P < 0.05, **P < 0.01, ***P < 0.001 (compared with the group treated with SR140333 alone).

We further explored the mechanisms of disruption of mitochondrial functions by NK-1R inhibition. Activation of NK-1R signaling has been linked to intracellular calcium mobilization (41), and calcium uptake into the mitochondrial matrix critically influences mitochondrial function (42, 43). We therefore analyzed intracellular calcium mobilization in response to modulation of NK-1R signaling activity using the cytosolic calcium indicator Fluo-4 AM and the mitochondrial calcium indicator Rhod-2 AM. Interestingly, stimulation of NK-1R signaling with SP generated only weak intracellular and mitochondrial calcium fluxes (*SI Appendix*, Fig. S8*A*). In contrast, application of SR140333 (Fig. 5 *A* and *B*) and Aprepitant (*SI Appendix*, Fig. S8 *B* and *C*) caused a rapid and transient cytosolic calcium elevation followed by a consistent rise of mitochondrial calcium concentration in both K562 and HL60 cells. H_2_O_2_ is an oxidative stress mediator that impairs mitochondrial structure and triggers apoptosis via calcium transfer between the ER and mitochondria (44). Using H_2_O_2_ as a positive control, we demonstrated that the cytoplasmic and mitochondrial calcium fluxes induced by SR140333 or Aprepitant were more potent and sustained than those induced by H_2_O_2_ (*SI Appendix*, Fig. S8*D*), strongly supporting calcium transfer from ER to mitochondria as an apoptosis stimulus.

**Fig. 6.**
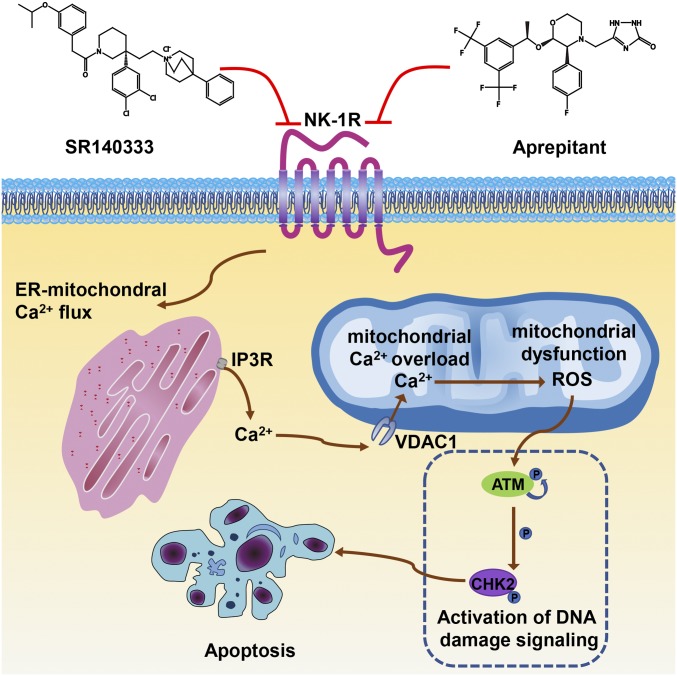
Schematic of blocking NK-1R induces apoptosis in human myeloid leukemia cells.

As calcium influx into mitochondria is known to contribute to pathological induction of cell death (45), we hypothesized that mitochondrial calcium overload caused mitochondrial dysfunction, contributing to ROS generation and cell apoptosis upon NK-1R inhibition. Indeed, calcium chelation with 1,2-Bis(*o*-aminophenoxy)ethane-*N*,*N*,*N*′,*N*′-tetraacetic acid (BAPTA) reduced mitochondrial ROS production by SR140333 (Fig. 5*C*) and increased cell viability (Fig. 5*D*). Similar results were also obtained following removal of calcium from the culture medium (*SI Appendix*, Fig. S9 *A* and *B*). The accumulation of calcium into the mitochondria depends on the ER, which serves as the main intracellular calcium storage organelle. Calcium can be released from the ER via inositol 1,4,5-triphosphate receptor (IP_3_R). The IP_3_R inhibitor 2-aminoethyl diphenylborinate (2-APB) reduced ROS production (Fig. 5*E*) and increased cell viability (Fig. 5*F*) in the presence of SR140333. The calcium released from the ER is transported to the mitochondria via voltage-dependent anion channel type 1 (VDAC1), which is located in the outer mitochondrial membrane. It is well recognized that VDAC1 is involved in many biological processes, including calcium homeostasis, energy metabolism, and apoptosis (46, 47). Pharmacological inhibition of VDAC1 by 4,4′-diisothiocyanostilbene-2,2′-disulfonic acid (DIDS) prevented SR140333-induced intracellular ROS generation (Fig. 5*G*) and cell death (Fig. 5*H*). Taken together, our results suggested blocking NK-1R with either Aprepitant or SR140333, 2 NK-1R antagonists with distinctive chemical structures, induced ER-mitochondrial calcium overload and, consequently, ROS accumulation and cell apoptosis (Fig. 6).

### Blocking NK-1R Alleviates Leukemia-Induced Bone Pain.

**Fig. 7.**
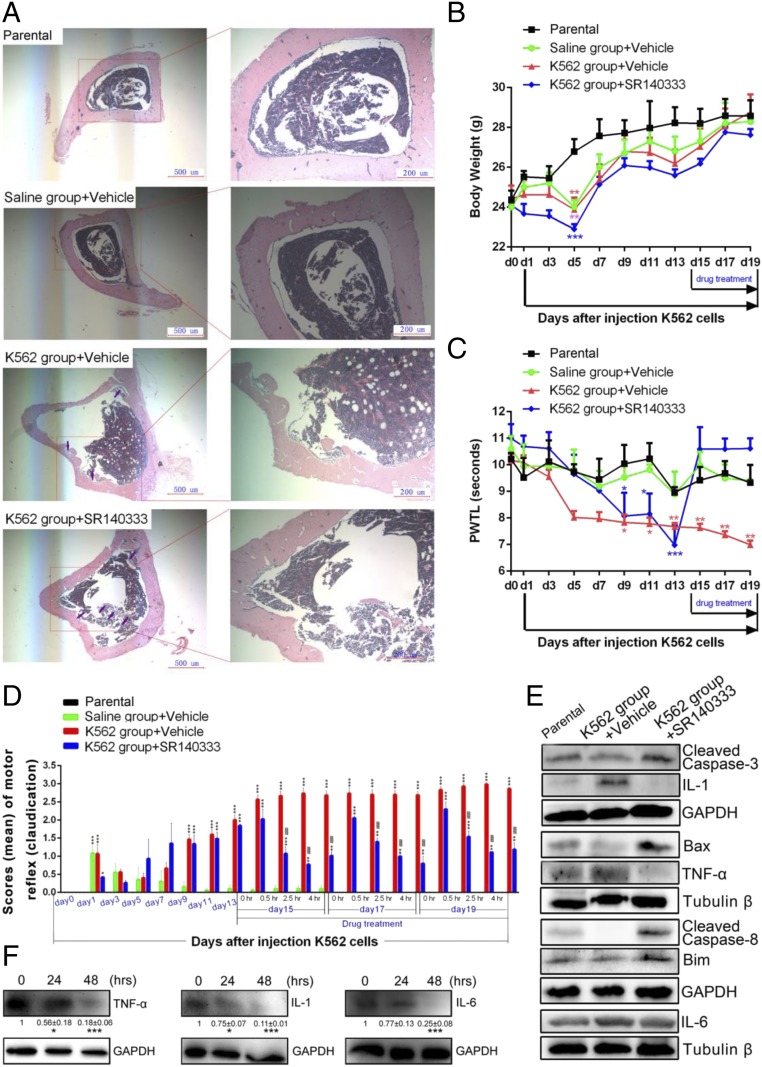
Blocking NK-1R alleviates leukemia-induced bone pain in vivo. (*A*) Photomicrographs of hematoxylin and eosin-stained sections of mouse tibial bone. The sections (4 μm) were taken from the tibial bone on day 21 after inoculation of K562 cells. (*B*) Changes of body weight. **P < 0.01, ***P < 0.001 (compared with the parental group on the same experiment day). (*C*) Measurement of PWTL in the hot plate tests. *P < 0.05, **P < 0.01, ***P < 0.001 (compared with the parental group on the same experiment day). Values represent mean ± SEM. (*D*) Spontaneous pain scores of motor reflex. Values represent mean ± SEM. *P < 0.05, **P < 0.01, ***P < 0.001 (compared with the saline group treated with vehicle on the same experiment day); ^###^*P* < 0.001 (compared with the K562 group treated with vehicle on the same experiment day). In *B*–*D*, *n* = 30 mice in each group. (*E*) Western blotting of the apoptosis and inflammation-related proteins in mouse bone marrow cells isolated from 15 mice of each group. GAPDH and Tubulin β were used as the loading controls. For the proteins probed in the same membrane (cleaved Caspase-3 and IL-1, Bax and TNF-α, cleaved Caspase-8 and Bim), 1 loading control was used. The protein quantification is shown in *SI Appendix*, Fig. S10*C*. (*F*) Western blotting of the inflammation-related proteins in K562 cells treated by SR140333 at 33 μM. Quantification of inflammation-related proteins by densitometry is shown. GAPDH was used as the loading control. Values are mean ± SEM (*n* = 3). *P < 0.05, ***P < 0.001 (compared with the control group).

We next explored the therapeutic potential of NK-1R antagonists in chronic pain of leukemia patients. To assess the analgesic effect of NK-1R antagonism, we exploited a bone pain mouse model involving intratibia injection of CML K562 cells that we have recently established (48). The sections obtained from the proximal end of the tibia at day 21 after cell injection showed that tumor transplantation caused various degrees of bone destruction, including irregular bone edges, bony cortex thinning, and cortex falling to the bone marrow cavity, which mimics the human situation (Fig. 7*A*). Consistent with our previous findings, body weight was decreased markedly within the first 4 d after K562 inoculation or saline injection, suggesting a stress response caused by experimental interruption (Fig. 7*B*). However, mice gradually gained body weight after day 5 postinoculation; after day 15, there was no significant difference between the 4 groups (Fig. 7*B*).

The hot plate and von Frey hair tests were used to assess the sensitivity of the mouse paw to thermal (Fig. 7*C*) and mechanical (*SI Appendix*, Fig. S10*A*) stimulation, respectively. The paw withdrawal thermal tendency (PWTL; Fig. 7*C*) and the paw mechanical withdrawal threshold (PMWT; *SI Appendix*, Fig. S10*A*) were decreased significantly 7 to 9 d after inoculation of tumor cells but were unaffected in the group injected with saline, consistent with our previous findings (48). Treatment with SR140333 rapidly restored both PMTL and PMWT values to the levels comparable to the parental group and the saline group. Similar findings were also observed in the spontaneous pain scoring (Fig. 7*D*) and the inclined-plane test, which evaluated muscle function and body balance behavior (*SI Appendix*, Fig. S10*B*).

Despite improved pain-related behaviors, we did not observe significant changes in bone destruction in the SR140333 treatment group compared with the vehicle group (Fig. 7*A*). Cancer-induced bone pain is correlated with a significant increase of proinflammatory mediators acting peripherally and centrally, contributing to neuronal hypersensitivity (49). We therefore examined the expression levels of tumor necrosis factor-α (TNF-α), interleukin-1 (IL-1), and IL-6, 3 key cytokines that contribute to cancer-induced bone pain. Indeed, the bone marrow cells from the mice transplanted with K562 cells expressed the cytokines at higher levels than the parental group, and treatment with SR140333 markedly reduced the abundance of these cytokines to a normal level (Fig. 7*E* and *SI Appendix*, Fig. S10*C*). We further examined the cytokine release in K562 cells upon exposure to SR140333 and demonstrated that SR140333 inhibited TNF-α, IL-1, and IL-6 expression in K562 cells (Fig. 7*F*). The cytotoxicity of SR140333 on the bone marrow cells was also evaluated. The mice inoculated with K562 cells showed decreased expression level of cleaved Caspase-3 and Caspase-8, Bax, and Bim compared with the parental group, and SR140333 treatment up-regulated the expression of these proapoptotic proteins compared with that of the parental and vehicle groups (Fig. 7*E* and *SI Appendix*, Fig. S10*C*). Therefore, our results suggested the analgesic effect of SR140333 in the CML-induced bone pain mouse model is associated with antiinflammatory effects in the bone/tumor microenvironment and the induction of leukemia cell apoptosis.

## Discussion

This study identified NK-1R as a target for treatment of human myeloid leukemia. Many NK-1R antagonists, including peptide and nonpeptide antagonists, have demonstrated good selectivity, potency, safety, and bioavailability in preclinical studies (8, 50). Aprepitant, an NK-1R antagonist in the clinic, is well tolerated with minimal side effects observed (24, 25), and thus can be directly used to test antitumor action in clinical trials. Besides cancer, the SP/NK-1R system plays an important role in pain transmission. Here, we demonstrate the proapoptotic and antinociceptive effect of NK-1R blockade in human myeloid leukemia cells and mouse models, implicating the potential multifactorial effects of NK-1R antagonists in human leukemia treatment.

This study further defined mitochondrial oxidative stress as a key factor contributing to the proapoptotic effect of NK-1R antagonists. Different from our finding in leukemia cells, an increase of ROS production following NK-1R activation was reported in immune cells (51), respiratory cells (52), and peripheral sensory neurons (38). Whether this discrepancy is associated with the fundamental differences between normal cells and tumor cells remains to be elucidated. Interestingly, despite a rapid reduction of mitochondrial oxidative phosphorylation and accumulation of mitochondrial ROS occurring as early as 3 h after blocking NK-1R (Fig. 4 *E* and *F*), SILAC-MS–based proteomic analysis revealed that a subset of mitochondria-related proteins, particularly the proteins involved in electron transport chain complexes, was up-regulated after 24 h (Fig. 4*D*). We speculate that a rapid mitochondrial dysfunction induced by blocking NK-1R causes an energy deficiency that results in a compensatory increase in energy production by increasing the abundance and activity of mitochondrial proteins. Therefore, the most likely explanation for the increase of electron transport chain protein expression is a compensatory reaction of the tumor cells in response to impaired mitochondrial function and energy deficiency.

The critical roles of metabolic adaption and reprograming in therapeutic response and the development of resistance have been increasingly acknowledged. Here, we demonstrated that NK-1R antagonists decreased OCR and ATP production by impairing mitochondrial functions via ER-mitochondrial calcium flux. Nevertheless, cancer cells may also respond to drug treatment by rewiring metabolic flux, for example, by regulating pathways that support increased glycolysis and/or glutamine utilization to provide alternative sources of ATP or strengthening the capability to detoxify mitochondrial-derived ROS (53). Cells that fail to metabolically adapt to the energetic and oxidative stresses will undergo cell death. For example, in response to metformin-mediated inhibition of oxidative phosphorylation, cancer cells enhance glycolysis and glutamine metabolism to restore cellular ATP levels, resulting in metformin resistance (54). Therefore, it will be of interest to investigate how metabolic rewiring affects the therapeutic response to NK-1R antagonists and whether targeting this metabolic vulnerability, for example, by combination with the drugs targeting mitochondria and increasing ROS (53, 55, 56), can improve the therapeutic efficacy.

We further revealed that increased ROS production upon blocking NK-1R is, at least in part, due to mitochondrial calcium overload. This induction of a calcium transient in the mitochondria after NK-1R blocking was entirely unexpected. As a GPCR, stimulation of NK-1R has been reported to cause intracellular calcium mobilization through activation of phospholipase C signaling. Indeed, we observed a moderate cytoplasmic calcium flux in response to SP stimulation, but with a much lower magnitude than blocking NK-1R by SR140333 (*SI Appendix*, Fig. S8*A*). Moreover, a rapid and remarkable mitochondrial calcium flux was detected upon SR140333 treatment but was undetectable following SP stimulation (*SI Appendix*, Fig. S8*A*). Our data suggested that the calcium signaling induced by NK-1R blocking differs from that following NK-1R activation, and thus mediates distinct biological consequences. The mechanisms underlying NK-1R antagonist-induced ER calcium release remain elusive. It is well established that GPCRs are synthesized and undergo processing and maturation in the ER (57). However, how GPCR signaling affects ER function is much less understood and will be investigated in our future studies. Overall, this mechanistic insight expands our understanding of NK-1R–mediated GPCR signaling in the regulation of intracellular calcium mobilization and mitochondrial redox homeostasis in cancer cells.

We also identified inhibition of mTORC1 signaling and MYC expression as acute outcomes of NK-1R inhibition (Fig. 4*B*). mTORC1 signaling is a master regulator of metabolism (9, 10, 18). However, a moderate cytostatic effect of mTORC1 inhibition did not support it as a key pathway mediating the cytotoxicity of NK-1R. Instead, it is most likely that decreased mTORC1 activity is the consequence of mitochondrial dysfunction and energy deficiency. MYC is another master regulator of metabolism (58). Whether a rapid decrease of MYC is associated with NK-1R inhibition–induced ROS production and cell apoptosis remains to be determined.

In addition to its potent anticancer activity, we identified the potential therapeutic application of NK-1R antagonists as analgesics to reduce bone pain of leukemia patients. We proposed that elevated SP and NK-1R expression in human CML cells stimulates cancer cell proliferation and release of cytokines into the tumor microenvironment. SP released by CML cells can also bind to NK-1R in endothelial cells of blood vessels in a paracrine manner, causing plasma extravasation and granulocyte infiltration (5), or can activate immune cells such as mast cells and neutrophils that express NK-1R (59), resulting in amplification of the inflammatory response. These secreted factors activate nociceptors on sensory neurons to transmit painful stimuli centrally.

Taken together, this study revealed antitumor effects of NK-1R antagonists, a class of antiemetic drugs used in conjunction with cancer chemotherapy to treat myeloid leukemia, which provides a therapeutic option for leukemia treatment. We also identified mitochondrial calcium overload-induced oxidative stress as a mechanism underlying the proapoptotic effect of NK-1R antagonists. This mechanistic finding expands our understanding of NK-1R–mediated GPCR signaling and facilitates the development of the next generation of NK-1R antagonists for cancer treatment.

## Materials and Methods

### AML Patients, Cell Lines, and Reagents.

Blood samples were collected from 25 healthy volunteers (Zhejiang Provincial Hospital of TCM) and 17 AML patients before receiving chemotherapy (Zhejiang Provincial Hospital of TCM and Zhejiang Province People’s Hospital). All of the participants signed the consent form. Any participant data or samples were deidentified before given to researchers. This study was approved by the Institutional Research Ethics Committee of Zhejiang Provincial Hospital of TCM.

Human normal CD34^+^ hematopoietic cells were collected from 5 healthy donors who were given 5 to 10 μg/kg of granulocyte-colony stimulating factor per day for 4 to 5 d at Zhejiang Provincial Hospital of TCM. Peripheral blood mononuclear cell collection was isolated by the COBE SPECTRA Apheresis System, followed by purification of CD34^+^ hematopoietic cells by a magnetic activated cell sorting system. The percentage of CD34^+^ hematopoietic cells was analyzed by flow cytometry.

Human myeloid leukemia cell lines K562, HL60, KG-1α, and NB4 were obtained from the Chinese Academy of Medical Sciences & Peking Union Medical College (generous gifts from Jingbo Zhang) and have been authenticated by single-nucleotide polymorphism array analysis prior to these studies. Details of the plasmid construction and transfection are provided in *SI Appendix*, *Supplemental Methods*.

SR140333 was synthetized by WuXi AppTec and dissolved in dimethyl sulphoxide (DMSO; Sigma). SP was synthetized by Sangon Biotech Co., Ltd. with >98% purity. DIDS and 2-APB were purchased from Sigma. Aprepitant was purchased from Solarbio. Trolox was purchased from Abcam. BAPTA-AM was purchased from TargetMol. MitoQ was purchased from MCE.

### Mouse Models and In Vivo Drug Studies.

Animal work was approved by the Ethics Committee of Animal Experiments at Zhejiang Sci-Tech University. K562 cells were implanted into the flanks of female BALB/c nude mice. When tumors reached 100 to 150 mm^3^, the mice were treated with SR140333 at a dose of 10 mg/kg (*n* = 7) or 0.6% DMSO in phosphate-buffered saline (*n* = 6) via in situ injection every day. The mice were euthanized when they reached the ethical end points, which are either tumor volume exceeding 1,600 cm^3^ or more than 20% weight loss.

A mouse model of leukemia-induced bone pain was established as previously reported (48). SR140333 at a dose of 5 mg/kg was injected in the mice of the experiment group at day 15, day 17, and day 19 by the tail intravenous route. The behavioral assays are fully described in *SI Appendix*, *Supplemental Methods*.

### Cell Proliferation Assay, ROS Detection, Calcium Mobilization Analysis, and Analysis of Bioenergetics Using the Seahorse XF96 Extracellular Flux Analyzer.

The number of viable cells was counted after Trypan blue staining. Mitochondrial ROS was detected by MitoSOX Red (Thermo Fisher Scientific). Calcium mobilization analysis was performed by staining cells with either Fluo-4 AM (Invitrogen) or Rhod-2 AM (Invitrogen) as described previously (60). All extracellular flux analyses were performed using the Seahorse Bioscience XF96 Extracellular Flux analyzer (Seahorse Bioscience). Detailed methods are available in *SI Appendix*, *Supplemental Methods*.

### SILAC Assay and RNA Sequencing.

K562 cells were labeled with “heavy isotopic lysine” (^13^C-Lysine) or “light isotopic lysine” (^12^C-Lysine) using a SILAC Protein Quantitation Kit (Thermo Scientific Pierce). The “light” labeled cells were then treated with 33 μM SR140333, and the “heavy” labeled cells were treated with the same volume of solvent in SILAC media for 24 h. Equal amounts of protein of the “light”-labeled cells and “heavy”-labeled cells were combined and subjected to high-performance liquid chromatography (HPLC) fractionation and quantitative proteomic analysis by liquid chromatography-tandem MS.

For RNA sequencing, the complementary DNA libraries were prepared as previously described (61) and sequenced on an Illumina HiSeq 2000 system (Illumina). The reads were mapped to the human reference genome hg38 assembly using TopHat-Cufflinks. Absolute gene expression was quantified by the fragments per kilobase per million. The sequences reported in this study have been deposited in the Sequence Read Archive database (accession no. PRJNA319129) (62).

### Statistical Tests.

The significance of difference of the means was tested by the 1-way ANOVA using SPSS software (version 19.0). Differences were considered significant when *P* < 0.05.

## Supplementary Material

Supplementary File

Supplementary File

Supplementary File
