# Supplementary material for: Neurokinin-1 receptor is an effective target for treating leukemia by inducing oxidative stress through mitochondrial calcium overload
Source: Proc Natl Acad Sci U S A. 2019 Sep 5;116(39):19635–45. doi: 10.1073/pnas.1908998116 (PMC6765257; doi:10.1073/pnas.1908998116)
Supplement: Supplementary File [file pnas.1908998116.sapp.pdf]

**Neurokinin-1 receptor is an effective target for treating leukemia by inducing oxidative stress through mitochondrial calcium overload**

Chentao Ge, Hemiao Huang, Feiyan Huang, Tianxin Yang, Tengfei Zhang, Hongzhang Wu, Hanwei Zhou, Qi Chen, Yue Shi, Yanfang Sun, Liangjue Liu, Xi Wang, Richard B. Pearson, Yihai Cao, Jian Kang, Caiyun Fu

Contents

Supplemental Methods……………………………………………………………...…2
Supplemental Figures…………………………………………………………...10
Figure S1 Immunocytochemical analysis of NK-1R and SP expression in peripheral blood samples of AML patients and healthy volunteers……………………………..10
Figure S2 Blocking NK-1R induced apoptosis in human myeloid leukemia cells..12
Figure S3 Quantification of apoptosis and cell cycle-related proteins by densitometry……………………………………………………………………………13
Figure S4 The effects of SR140333 on the proliferation of normal mouse bone marrow cells and the rate of hemolysis in human red blood cells…………………...14
Figure S5 The effects of Everolimus on cell viability……………………………15
Figure S6 Blocking NK-1R up-regulated the expression levels of proteins in mitochondrial oxidative phosphorylation complex…………………………………..16
Figure S7 Blocking NK-1R induced mitochondrial oxidative stress……………...17
Figure S8 Intracellular calcium mobilization in response to NK-1R activation or inhibition……….........................................................................................................19
Figure S9 Mitochondrial calcium flux contributes to oxidative stress and cell death in response to NK-1R inhibition……………………………………………………..21
Figure S10 Blocking NK-1R alleviated leukemia-induced bone pain in vivo…….22
Supplemental Table 1. The information of myeloid leukemia patients……………...24
Reference……………………………………………………………………………..25



www.pnas.org/cgi/doi/10.1073/pnas.1908998116

## Supplemental methods

*AML patients*

Blood samples were collected from 25 healthy volunteers (Zhejiang Provincial Hospital of TCM) and 17 AML patients before receiving chemotherapy (Zhejiang Provincial Hospital of TCM and Zhejiang Province People's Hospital). All the participants have signed the consent forms. Any participant data or samples were de-identified before given to researchers. This study was approved by the Institutional Research Ethics Committee of Zhejiang Provincial Hospital of TCM. The AML patient information including age, sex, subtype and peripheral WBC count were provided in Supplementary Table S1.

Human normal CD34+ hematopoietic cells were collected from 5 healthy donors who were given 5-10 μg/kg/day of G-CSF for 4-5 days in Zhejiang Provincial Hospital of TCM. Peripheral blood MNC collection was isolated by the COBE SPECTRA Apheresis System followed by purification of CD34+ hematopoietic cells by a magnetic activated cell sorting system. The percentage of CD34+ hematopoietic cells was analyzed by flow cytometry.

*Cell lines*

Human myeloid leukemia cell lines K562, HL60, KG-1α and NB4 were obtained from the Chinese Academy of Medical Sciences & Peking Union Medical College (generous gifts from Professor Jingbo Zhang). These cell lines have been screened for mycoplasma contamination using Mycoplasma Stain Assay Kit (Beyotime Biotechnology, Shanghai), and authenticated by SNP array analysis prior to these studies. K562 cells were cultured in DMEM (Life Technologies, Carlsbad, USA) and HL60 cells were cultured in RPMI-1640 (Life Technologies, Carlsbad, USA), supplemented with 10% heat-inactivated fetal bovine serum (FBS, Sijiqing Biotechnology Co., China).

*Reagents*

SR140333 was synthetized by WuXi AppTec (China) and dissolved in DMSO (dimethyl sulphoxide, Sigma) (Fu et al., 2008). MitoQ (Cat#HY-100116) was purchased from MCE, Aprepitant (Cat# YZ-1041904) was purchased from Solarbio,

Trolox (Cat# ab120747) was purchased from Abcam, 1,2-Bis(2-aminophenoxy)ethane-N,N,N′,N′-tetraacetic acid tetrakis (acetoxymethyl ester) (BAPTA-AM, Cat T6245), was purchased from TargetMol. 4,4'-diisothiocyanostilbene-2,2'-disulfonic acid (DIDS, Cat# D3514) and 2-Aminoethoxydiphenyl borate (2-APB, Cat# D9754) were purchased from Sigma.

*K562 human chronic myeloid leukemia xenograft*

Animal work was approved by the Ethics Committee of Animal Experiments at Zhejiang Sci-Tech University. Female BALB/c nude mice of 5 weeks old were purchased from Shanghai SLAC laboratory animal center. K562 cells were implanted into the flanks of female BALB/c nude mice. When tumors reached 100–150 $mm^3$, mice were treated with SR140333 at a dose of 10 mg/kg (n = 7) or 0.6% DMSO in PBS (n = 6) via *in situ* injection every day. The tumor volumes and body weights were measured every 2 days (Lu et al., 2016). The mice were euthanized when they reached the ethical endpoints which are either tumor volume exceeded 1600 $cm^3$, or more than 20% weight loss.

*Estabolishment of a mouse model of leukemia-induced bone pain and behavioral assays for the assessment of the leukemia-induced bone cancer pain*

A mouse model of leukemia-induced bone pain was established as previously reported (Zhu et al., 2015). Female ICR mice obtained from Hangzhou Normal University, China, were randomly divided into 4 groups (30 mice per group) including parental group (control group 1), saline group treated with vehicle (control group 2), K562 group treated with vehicle (control group 3), and K562 group treated with SR140333 (experiment group). On day 0, the K562 cells or saline were injected into the mouse tibial bone marrow cavity of the left leg. SR140333 at a dose of 5 mg/kg was injected in the mice of experiment group at day 15, day 17 and day 19, by the tail intravenous route. Control groups 2 and 3 were treated with 0.6% DMSO in PBS.

Before each test, the mice were allowed to acclimatize for at least 30 min. In addition to behavioral measurements mentioned below, each mouse was monitored for changes in body weight, diet, growth performance and activities. Behavioral measurements and body weight measurements were performed on day 0 before inoculation and on post-inoculation day 1, 3, 5, 7, 9, 11, 13, 15, 17 and 19. The behavioral tests were performed by the researchers who were blind to the experimental conditions. All tests were performed during the light phase.

Mechanical allodynia was measured by the hind paw mechanical withdrawal threshold (PMWT) response to von Frey filament (Stoelting, Wood Dale, Illinois, USA) stimulation according to the method originally described by Kim and Chung (Kim and Chung, 1992). Mechanical stimulation was carried out with von Frey filaments with logarithmically incremental stiffness that delivers force to the hind paw measured in grams (range: 0.16–6.0 g). The test environment consisted of a L15×W10.7×H13.8 $cm^3$ perspex box situated on a wire mesh platform. During testing the mouse was placed in the test box and allowed to settle for 10 min. Fifty percent paw withdrawal threshold was determined using the up-down testing paradigm (Chaplan et al., 1994). Testing was initiated with the 0.4 g filament. Whenever a positive response to a stimulus occurred, the next lower von Frey hair was applied, and whenever a negative response occurred, the next higher hair was applied. The testing consisted of five more stimuli after the first change in response occurred, and the pattern of response was converted to a 50% von Frey threshold using the method described by Kim and Chung (Kim and Chung, 1992).

For the hot plate test to evaluate the paw withdrawal thermal latency (PWTL), mice were placed on a hot plate of hot/cold plate analgesia instrument (YLS-21A, Jinan, Shandong, China) adjusted to 55°C (thermal hyperalgesia). The latency of the first reaction was recorded (licking the paws or jumping) with a cutoff time of 30 s (Zhu et al., 2015).

For spontaneous pain scoring, mice were placed in a large plastic observation box with the smooth floor as described previously (Zhu et al., 2015). Briefly, scores were given as following: (0) free movement, the activity of treated limb is as same as that of the control limb, (1) indicates slight limp, (2) indicates extent between (1) and (3), (3) indicates severe limp, and (4) indicates complete lack of limp.

Inclined-plate test was performed to assess muscular strength and the proprioception according to the publications (Ou et al., 2011; Zhu et al., 2015). In brief, the mouse was placed crosswise to the long axis of an inclined plate with an initial angle of 30°. The angle was then adjusted in 2-degree increments. The maximum angle of the plate on which the mouse maintained its body position for 5 s without falling was determined. Each mouse was measured three times and the average value from three measurements was set as the inclined-plane degree.

*shRNA constructions and transfection*

Two different small hairpin RNAs (shRNAs) against NK1R were designed. NK1R-shRNA (NK1R-shRNA1 and NK1R-shRNA2) constructs were produced as described previously(Fang et al., 2012). The sequences of the oligonucleotides homologous to a 21-nucleotide segment of NK1R were: 5’-GCCAGUAUCUACUCCAUGAUU-3’ for NK1R-shRNA1 and 5’-CCUACAUCAACCCAGAUCUUU-3’ for NK1R-shRNA2. A scramble shRNA

(negative control, Con-shRNA) was also designed. For transfection, plasmids (2.5 μg) were transfected into K562 cells using Liposome reagent (Hanbio technology Co., Ltd) according to the manufacturer's protocol. For a period of time after transfection, appropriate concentrations of puromycin were added for screening and stable cell lines were used for further studies.

*Cell proliferation assay*

Cells were seeded in a 48-well plate at a density of $1\times10^4$ cells/well. The cell suspension after staining with 0.02% trypan blue (Solarbio) was collected at indicated time points to measure the numbers of live cells by trypan blue exclusion assay using cell counter (Countstar). The cell viability was calculated as the percentage of live cells in drug treated group relative to the vehicle treated group.

*ROS detection*

MitoSOX Red (5 μM; Thermo Fisher, USA) was added to cell culture and incubated at 37°C for 30 min before analysis by FACSAria.

*Calcium mobilization analysis*

Cells were plated in cover glass-bottom dish (SPL, Korea) and stained with either Fluo-4 AM (5 μM; Invitrogen) or Rhod-2 (2 μM; Invitrogen) for 30 minutes before analysis by a laser scanning confocal microscope (Nikon, Inc., Tokyo, Japan). After the initial measurement for 50 seconds to determine the baseline fluorescence, SR140333 at 60 μM for K562 cells or 30 μM for HL60, was added. Aprepitant at 40 μM for both K562 and HL60 was added. Image acquisition continued for 1500 to 2500 seconds after drug treatment. Calcium concentrations were expressed as the average fluorescence intensity of 20 cells/field randomly from at least three fields at each time point as described previously (Lu et al., 2016).

*Analysis of bioenergetics using the Seahorse XF96 extracellular flux analyzer*

All extracellular flux analyses were performed using the Seahorse XF96 extracellular flux analyzer (Seahorse Bioscience, Billerica, USA). Cells were treated with SR140333 at the indicated concentrations or vehicle for three hours. Prior to the assay, cells were washed with assay running buffer (unbuffered DMEM containing 5 mM

glucose, 1 mM glutamine and 1 mM sodium pyruvate) and seeded at $2\times10^4$ cells in the running buffer. The cells were equilibrated for 30 minutes at 37°C. Compounds (Oligomycin 1 μM, FCCP 1.5 μM, Rotenone 0.5 μM and Antimycin 0.5 μM) were injected during the assay and OCR was measured using 2 min measurement periods. At the completion of each assay, the cells were stained with Hoechst 10 μM. Images were acquired in Cellomics Cellinsight 1 and analyzed by the software to determine the cell number per well.

*Immunocytochmistry of blood samples of acute myeloid leukemia patients*

The peripheral blood smears of AML patients or healthy voluteers were fixed in 10% buffered formalin for 40 minutes at 4°C. After fixation, the slides were washed in PBS (pH 7.4) for 3 times and then were treated with 0.25% Triton X-100 in PBS for 20 minutes at 4°C. After blocking with 10% goat serum, the slides were incubated with a NK-1R antibody (1:200, OmnimAbs, OM265084), SP antibody (1:200, Bioworld, BS1598), or a negative control mouse IgG at 4°C overnight. Subsequently, the slides were incubated with a rabbit anti-mouse IgG conjugated to HRP (1:500, Bioworld, BS13278) at room temperature for 1 hour prior to coloration with 3,3-diaminobenzidine (DAB) for 5 minutes at room temperature. The images of 20 different visual fields at X1000 magnification were taken by an optical microscope (OLYMPUS, BX53) for each slide. Two pathologists evaluated the results independently. The intensity of staining was assessed on a graded scale (0=negative; 1= weak positive; 2=moderate positive and 3=strong positive).

*Hematoxylin and Eosin (H&E) staining*

To assess the effect of SR140333 in the mouse model of leukemia-induced bone pain, SR140333 at a dose of 5 mg/kg or 0.6%DMSO in PBS was injected in the mice of experiment group (K562 group + SR140333) and control groups (saline group + vehicle or K562 group + vehicle), respectively, at day 15, day 17, day 19 and day 21 by tail intravenous route after cell inoculation at day 0. The method of establishing the mouse model of leukemia-induced bone pain and drug treatment has been fully described in the section of Materials and Methods. On day 21, 15 mice per group were sacrificed 2.5 hours after drug treatment for histopathological study and the other 15 mice per group sacrificed for isolation of bone marrow cells and protein extraction. For the histopathological study, left tibia was taken, fixed in 10% formalin solution for 24 hours, and dehydrated by ethanol with increasing concentrations. 10% EDTA (pH7.4) was used as the solution for decalcifying bone material for traditional paraffin section and H&E staining. After processing, the tissues were embedded in paraffin and sectioned to a thickness of 4 μm using a histocut (Leica, RM2235, Germany). Stained sections with H&E staining were observed by light microscopy

(NIKON TE2000-U). Image Pro Plus 6.0 software was used for the analysis of immunohistochemical results.

*Western blotting*

The human white blood cells were isolated from heparinized peripheral blood using red blood cell lysis buffer (Solarbio, China) and then lysed in RIPA lysing solution (Beyotime Biotechnology, China) before applied to a gel electrophoresis.

To prepare protein lysates of cultured cells, K562 or HL60 cells were collected from the culture plates and the mouse bone marrow cells from both femur and tibia were flushed out with Dulbecco's modified medium (DMEM) (Life Technologies) using a syringe into a sterile tube. Cells were washed with PBS three times and then lysed in the RIPA lysis buffer (1% Triton X-100, 1% deoxycholate, 0.1% SDS) containing 1 mM PMSF. Protein concentrations were determined using the BCA protein assay kit (Solarbio, PC0020) according to the manufacturer's instructions. Lysate containing 20-40 μg protein was subjected to SDS-PAGE and then transferred to PVDF membrane (Millipore Corp., Billerica, MA). The primary antibodies used were listed below. Bands were quantified using Image J and target protein intensities were normalized to the loading control for each sample.

| Protein | Brand | Catalogue | Dilution | Source |
|---|---|---|---|---|
| **4EBP1** | Cell signaling | 9452 | 1:2000 | rabbit |
| **P-4EBP1(S65)** | Cell signaling | 9451 | 1:1000 | rabbit |
| **ACTIN** | MPBio | MPBio-691001 | 1:10000 | mouse |
| **AKT** | Cell signaling | 4691 | 1:2000 | rabbit |
| **P-AKT (S473)** | Cell signaling | 4058 | 1:2000 | rabbit |
| **ATM** | GeneTex | GTX70103 | 1:1000 | mouse |
| **P-ATM(S1981)** | Cell signaling | Ab81292 | 1:1000 | Rabbit |
| **BCL-2** | Bioworld | BS1511 | 1:5000 | rabbit |
| **BCL-xL** | Bioworld | BS1032 | 1:1000 | rabbit |
| **BAX** | Bioworld | BS2538 | 1:1000 | rabbit |
| **BIM** | Bioworld | BS1035 | 1:500 | rabbit |
| **CDK4** | Bioworld | BS1392 | 1:1000 | rabbit |
| **Cdc25A** | Bioworld | BC1831 | 1:1000 | rabbit |
| **CHK1** | Santa Cruz | Sc7898 | 1:1000 | rabbit |
| **P-CHK1(S345)** | Cell signaling | 2348 | 1:1000 | rabbit |
| **CHK2** | Cell signaling | 6334 | 1:2000 | rabbit |
| **P-CHK2(T68)** | Cell signaling | 2197 | 1:1000 | rabbit |
| **Cleaved Caspase 3** | Bioworld | ARG54938 | 1:1000 | rabbit |
| **Cleaved Caspase 8** | Bioworld | AP0358 | 1:1000 | rabbit |
| **Cleaved Caspase 9** | Bioworld | BS1388 | 1:800 | rabbit |

| | | | | |
|---|---|---|---|---|
| **COX17** | Bioworld | BS2514 | 1:1000 | rabbit |
| **Cyclin D1** | Bioworld | BS1741 | 1:800 | rabbit |
| **Cyclin B1** | Bioworld | BS1392 | 1:500 | rabbit |
| **ERK1/2** | Cell signaling | 9102 | 1:2000 | rabbit |
| **P-ERK1/2(T202/Y204)** | Cell signaling | 9101 | 1:2000 | rabbit |
| **GAPDH** | Bioworld | AP0063 | 1:10000 | rabbit |
| **gamaH2AX(S139)** | Abcam | Ab81299 | 1:1000 | rabbit |
| **IL-1** | Bioworld | BS2825 | 1:1000 | rabbit |
| **IL-6** | Bioworld | BS6419 | 1:1000 | rabbit |
| **MYC** | abcam | Ab32072 | 1:1000 | rabbit |
| **NDUFA8** | Bioworld | BS3336 | 1:1000 | rabbit |
| **NDUFB8** | abcam | Ab110242 | 1:2000 | mouse |
| **NK-1R** | Bioworld | BS2632 | 1:1000 | rabbit |
| **p16** | Bioworld | BS1265 | 1:500 | rabbit |
| **p21** | Bioworld | AP0713 | 1:800 | rabbit |
| **p65** | Cell signaling | 8242 | 1:1000 | rabbit |
| **P-p65(S536)** | Cell signaling | 3033 | 1:1000 | rabbit |
| **PARP** | Cell signaling | 9532 | 1:100 | rabbit |
| **S6RP** | Cell signaling | 2217 | 1:2000 | rabbit |
| **P-S6RP** | Cell signaling | 2215 | 1:2000 | rabbit |
| **SP** | Bioworld | AP6027 | 1:1000 | rabbit |
| **TNF-α** | Bioworld | ARG10158 | 1:500 | rabbit |
| **Tubulin β** | Bioworld | AP0064 | 1:10000 | rabbit |

*SILAC assay*

K562 cells were cultured in DMEM medium supplemented with 10% FBS and "heavy isotopic lysine" ($^{13}$C-Lysine) or "light isotopic lysine" ($^{12}$C-Lysine) using a SILAC Protein Quantitation Kit (Pierce, Thermo) for more than six generations. The cells were further expanded in SILAC media to desired cell number (approximately $5\times10^8$). The "light" labeled cells were then treated with 33 μM SR140333 and the "heavy" labeled cells were treated with the same volume of solvent in SILAC media for 24 hours. Equal amounts of protein of the "light" labeled cells and "heavy" labeled cells were combined and subjected to HPLC fractionation and quantitative proteomic analysis by LC-MS/MS.

*RNA sequencing*

K562 cells were treated with SR140333 at 33 μM for 12 hours followed by RNA extraction. The cDNA libraries were prepared as previously described (Ren et al., 2012) and sequenced on Illumina HiSeq 2000 (Illumina). After the base calling, the reads with either adaptor sequences, or low-quality reads which have more than 10% bases with QA less than 20 or more than 10% "N" bases, were removed. The reads

with high-quality scores were mapped to the human reference genome hg38 assembly using tophat-cufflinks. Absolute gene expression was quantified by the Fragments per Kilobase per Million (FPKM). The sequences reported in this study have been deposited in the Sequence Read Archive database with accession number PRJNA319129.

**Supplemental Figures**

Supplemental Figure 1

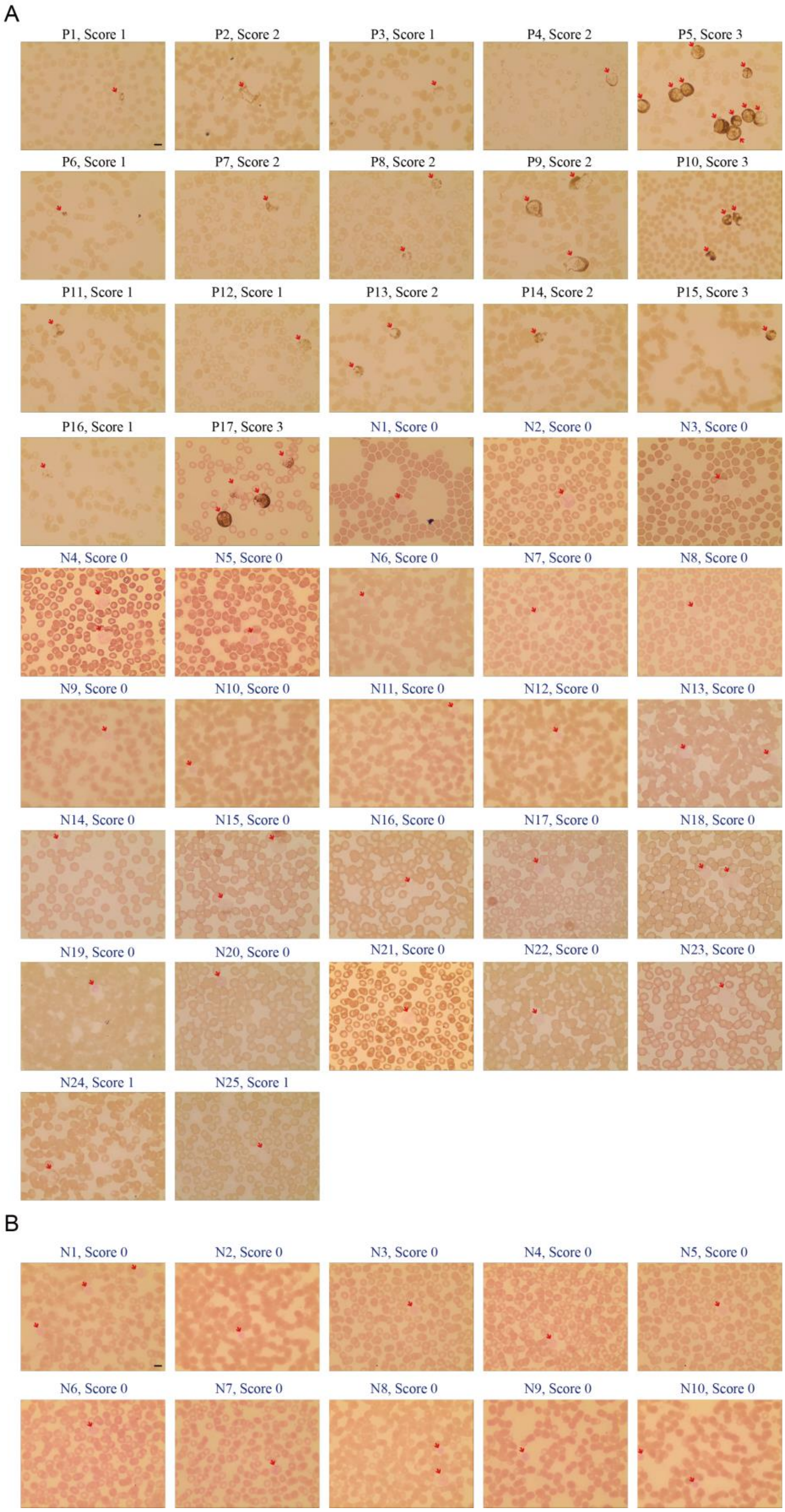

**Figure S1 Immunocytochemical analysis of NK-1R and SP expression in human peripheral blood samples.** (A) NK-1R expression of 17 AML patients (P) and 25 normal healthy volunteers (N). (B) SP expression of 10 normal healthy volunteers (N). The intensity of positively stained cells was scored using a scale of 0 (negative), 1 (weak), 2 (moderate) and 3 (strong). Scale bar represents 20 μM.

Supplemental Figure 2

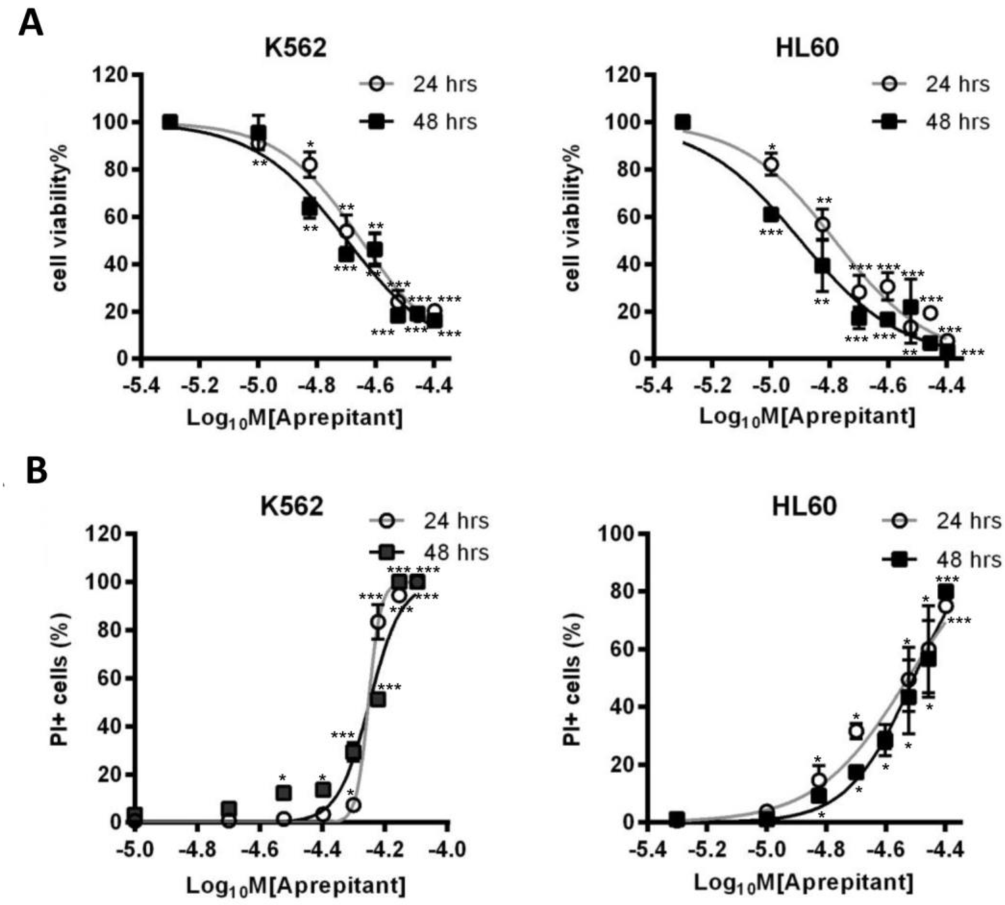


**Figure S2 Blocking NK-1R induces apoptosis in human myeloid leukemia cells**. (A) Cell viability after treatment with Aprepitant at the indicated concentrations for 24 hours and 48 hours. Values represent means ± SEM (n = 3). (B) The percentage of PI-positive cells after treatment with Aprepitant at the indicated concentrations for 24 hours and 48 hours. Values represent means ± SEM (n = 3). *$P$<0.05, ** $P$<0.01, ***$P$<0.001, compared with the vehicle treated cells.

Supplemental Figure 3

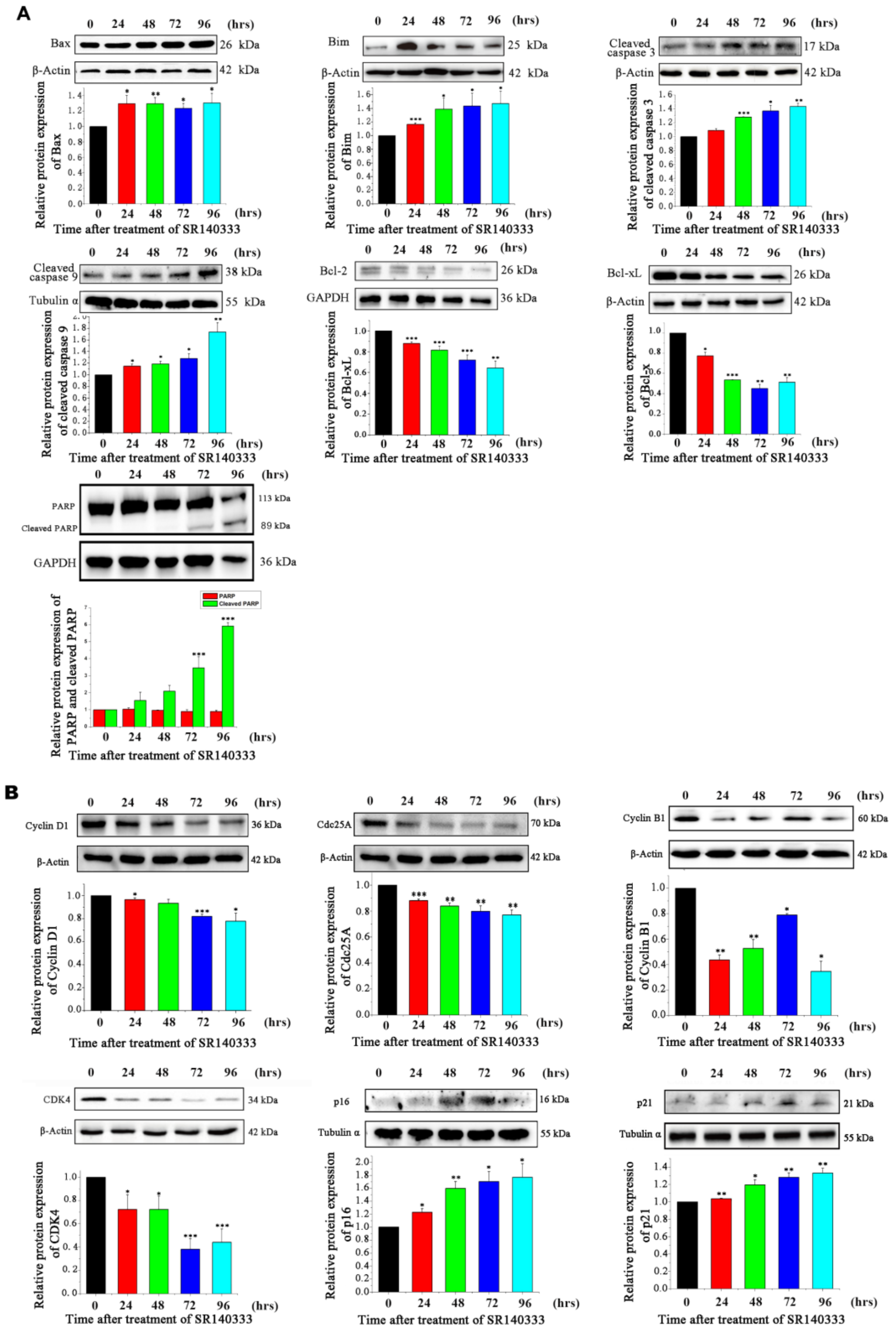


**Figure S3 Quantification of apoptosis (A) and cell cycle-related proteins (B) by densitometry.** GAPDH, Tubulin α and β-actin were used as the loading controls. For the proteins probed in the same membrane (Cyclin D1 and Cdc25A), one loading control was used. **P*<0.05, ** *P*<0.01, ****P*<0.001, compared with the sample at 0 hr time point as the control group.

Supplemental Figure 4

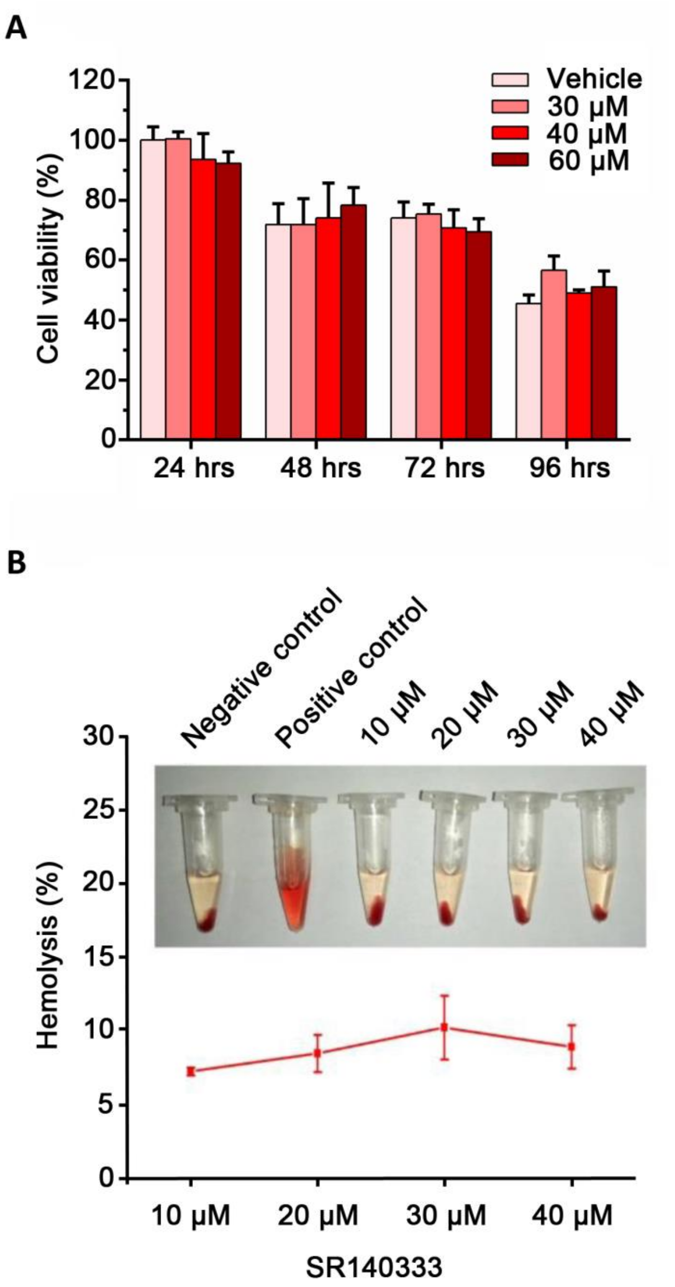


**Figure S4 The effects of SR140333 on the proliferation of normal mouse bone marrow cells and the rate of hemolysis in human red blood cells.** (A) Relative cell number of normal mouse bone marrow cells after exposure to various concentrations of SR140333 at the indicated time points (n=3). (B) The relative rate of hemolysis in human RBCs after incubation with SR140333 at the indicated concentrations for 24 hours. The positive control group was treated with 50 μl of 2% (v/v) Triton X-100 and the negative control group treated with 50 μl of PBS.

Supplemental Figure 5

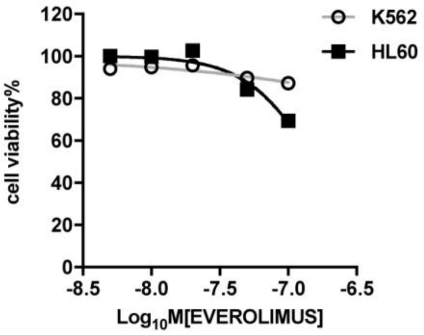


**Figure S5 The effect of Everolimus on cell viability.** Cells were treated with Everolimus at the indicated concentrations for 48 hours. The cell viability was calculated as the percentage of live cells in Everolimus treated group relative to the vehicle treated group. The live cells were counted by trypan blue exclusion.

Supplemental Figure 6

**A**

| Gene | ID | Protein name | L/H ratio |
|---|---|---|---|
| **oxidative phosphorylation complex I** | | | |
| MT-ND1 | P03886 | NADH-ubiquinone oxidoreductase chain 1 | 2.03 |
| MT-ND3 | P03897 | NADH-ubiquinone oxidoreductase chain 3 | 3.76 |
| NDUFS1 | P28331 | NADH-ubiquinone oxidoreductase 75 kDa subunit, mitochondrial | 1.33 |
| NDUFS2 | O75306 | NADH dehydrogenase [ubiquinone] iron-sulfur protein 2, mitochondrial | 1.46 |
| NDUFS3 | O75489 | NADH dehydrogenase [ubiquinone] iron-sulfur protein 3, mitochondrial | 1.54 |
| NDUFS4 | O43181 | NADH dehydrogenase [ubiquinone] iron-sulfur protein 4, mitochondrial | 1.84 |
| NDUFS8 | O00217 | NADH dehydrogenase [ubiquinone] iron-sulfur protein 8, mitochondrial | 1.44 |
| NDUFV1 | P49821 | NADH dehydrogenase [ubiquinone] flavoprotein 1, mitochondrial | 1.33 |
| NDUFV2 | P19404 | NADH dehydrogenase [ubiquinone] flavoprotein 2, mitochondrial | 1.53 |
| NDUFA2 | O43678 | NADH dehydrogenase [ubiquinone] 1 alpha subcomplex subunit 2 | 1.68 |
| NDUFA6 | P56556 | NADH dehydrogenase [ubiquinone] 1 alpha subcomplex subunit 6 | 1.43 |
| NDUFA7 | O95182 | NADH dehydrogenase [ubiquinone] 1 alpha subcomplex subunit 7 | 1.59 |
| NDUFA8 | P51970 | NADH dehydrogenase [ubiquinone] 1 alpha subcomplex subunit 8 | 1.43 |
| NDUFA9 | Q16795 | NADH dehydrogenase [ubiquinone] 1 alpha subcomplex subunit 9, mitochondrial | 1.55 |
| NDUFA11 | Q86Y39 | NADH dehydrogenase [ubiquinone] 1 alpha subcomplex subunit 11 | 1.68 |
| NDUFA13 | Q9P0J0 | NADH dehydrogenase [ubiquinone] 1 alpha subcomplex subunit 13 | 1.49 |
| NDUFB3 | O43676 | NADH dehydrogenase [ubiquinone] 1 beta subcomplex subunit 3 | 1.59 |
| NDUFB4 | O95168 | NADH dehydrogenase [ubiquinone] 1 beta subcomplex subunit 4 | 1.75 |
| NDUFB5 | O43674 | NADH dehydrogenase [ubiquinone] 1 beta subcomplex subunit 5, mitochondrial | 1.54 |
| NDUFB6 | O95139 | NADH dehydrogenase [ubiquinone] 1 beta subcomplex subunit 6 | 1.46 |
| NDUFB7 | P17568 | NADH dehydrogenase [ubiquinone] 1 beta subcomplex subunit 7 | 1.52 |
| NDUFB8 | O95169 | NADH dehydrogenase [ubiquinone] 1 beta subcomplex subunit 8, mitochondrial | 2.39 |

| Gene | ID | Protein name | L/H ratio |
|---|---|---|---|
| **oxidative phosphorylation complex II** | | | |
| SDHC | Q99643 | Succinate dehydrogenase cytochrome b560 subunit, mitochondrial | 1.6 |
| **oxidative phosphorylation complex IV** | | | |
| COX4I1 | P13073 | Cytochrome c oxidase subunit 4 isoform 1, mitochondrial | 1.38 |
| COX5B | P10606 | Cytochrome c oxidase subunit 5B, mitochondrial | 1.63 |
| COX17 | Q14061 | Cytochrome c oxidase copper chaperone | 1.33 |
| **oxidative phosphorylation complex V** | | | |
| ATP5I | P56385 | ATP synthase subunit e, mitochondrial | 1.57 |
| ATP5J | P18859 | ATP synthase-coupling factor 6, mitochondrial | 1.32 |
| ATP5O | P48047 | ATP synthase subunit O, mitochondrial | 1.37 |
| ATP6V0A2 | Q9Y487 | V-type proton ATPase 116 kDa subunit a isoform 2 | 1.32 |
| MT-ATP8 | P03928 | ATP synthase protein 8 | 1.92 |

**B**

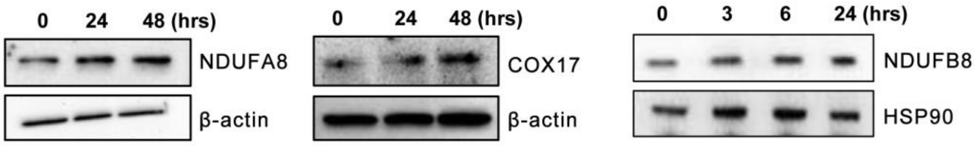


**Figure S6 Blocking NK-1R up-regulated the expression levels of proteins in mitochondrial oxidative phosphorylation complexes.** (A) The list of proteins in oxidative phosphorylation complexes whose expression levels were up-regulated upon SR140333 treatment based on SILAC quantitative proteomic profiling. (B) Western blotting of electron transport chain proteins in K562 cells treated with 33 μM SR140333 at the indicated time points. β-actin or HSP90 was used as the loading control.

Supplemental Figure 7

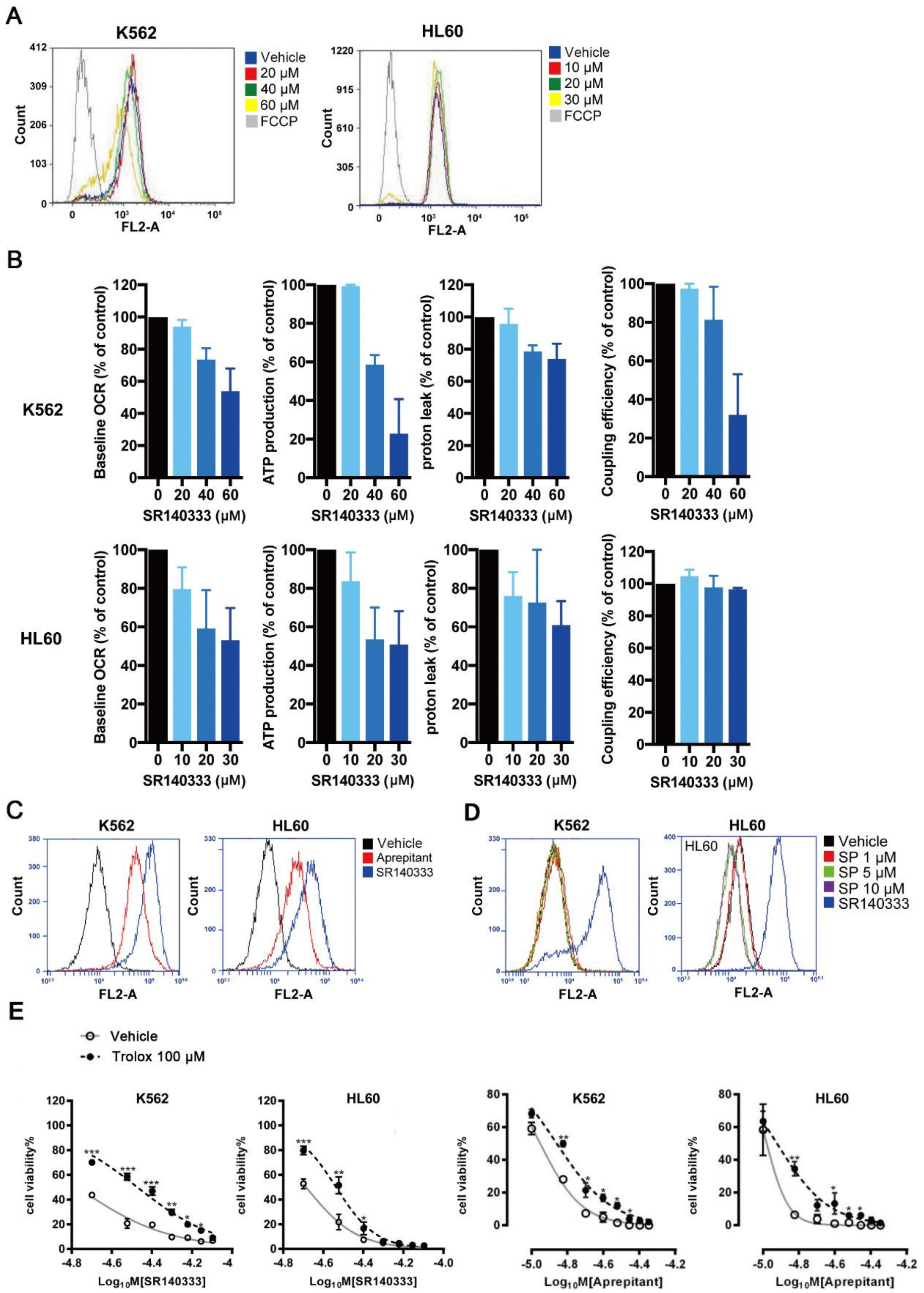

**Figure S7 Blocking NK-1R induced mitochondrial oxidative stress.** (A) The effect of SR140333 treatment for three hours on mitochondrial membrane potential measured by TMRE staining. (B) K562 and HL60 cells were treated with SR140333 at the indicated concentrations for three hours. Mitochondrial basal OCR, ATP production, proton leak, and coupling efficiency were measured by the Seahorse Bioscience XF96 extracellular flux

analyzer (n=3). (C) The effect of Aprepitant (24 μM and 19 μM for K562 and HL60, respectively) and SR140333 (60 μM and 40 μM for K562 and HL60, respectively) for three hours on mitochondrial ROS production measured by MitoSOX. (D) The effect of SP stimulation for three hours on cellular ROS production measured by MitoSOX. (E) K562 and HL60 cells were pre-treated with Trolox (100 μM) for one hour, and then treated with SR140333 or Aprepitant at the indicated doses for 24 hours. The cell viability was calculated as the percentage of live cells in the drug treatment group relative to the vehicle group. The live cells were counted by trypan blue exclusion. *$P$<0.05, ** $P$<0.01, ***$P$<0.001, compared with the group untreated with Trolox or Aprepitant.

Supplemental Figure 8

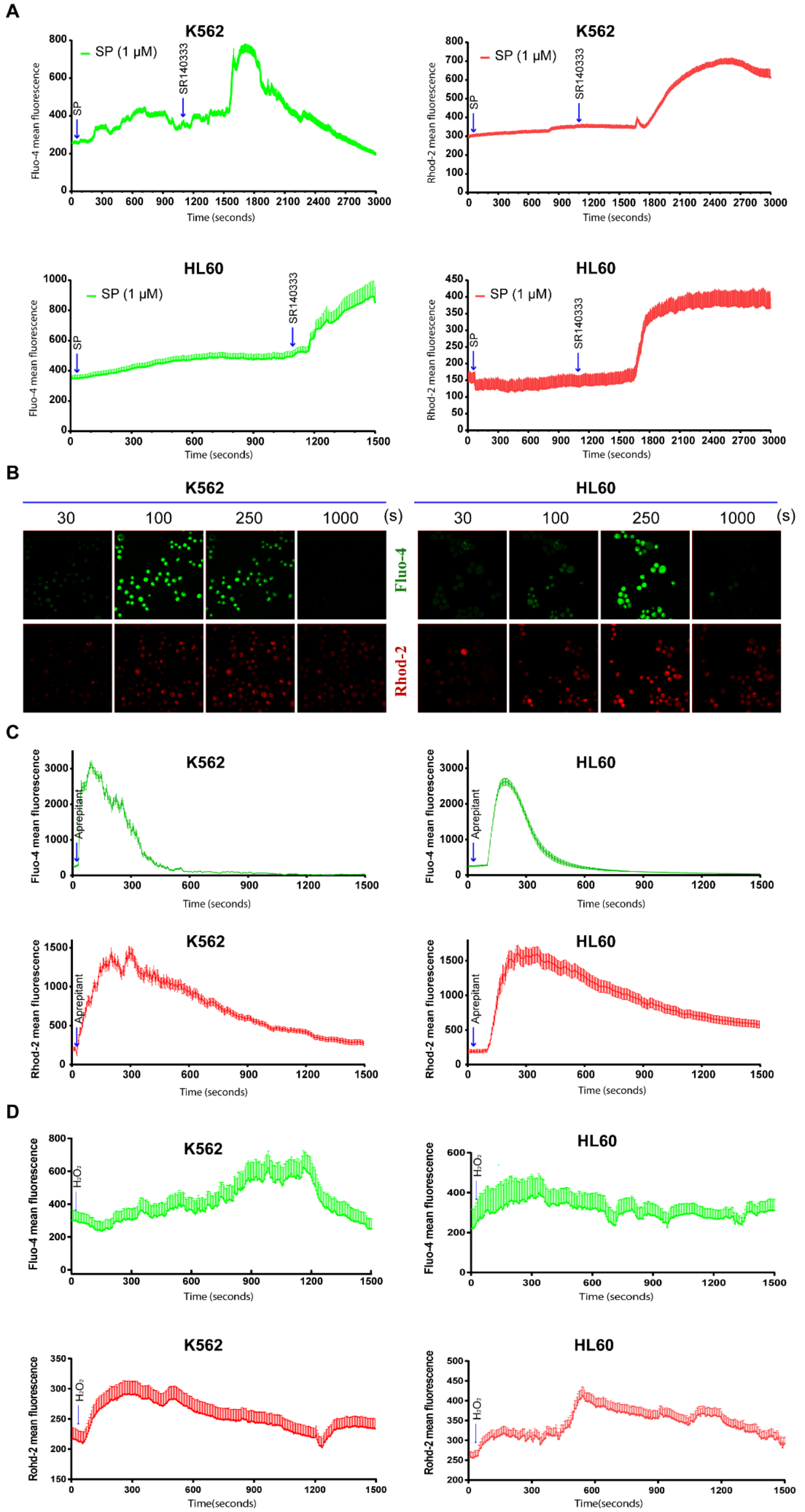

**Figure S8 Intracellular calcium mobilization in response to either NK-1R activation or inhibition.** (A) Quantitative results of cytosolic calcium indicator Fluo-4 fluorescence intensity (green) and mitochondrial calcium indicator Rhod-2 (red) fluorescence intensity in K562 and HL60 cells upon treatment with SP followed by SR140333. The doses of SR140333 were 60 μM and 30 μM for K562 and HL60, respectively. (B) Images of cytosolic calcium indicator Fluo-4 AM staining and mitochondrial calcium indicator Rhod-2 staining in K562 and HL60 cells. After the initial measurement for 50 seconds to determine the baseline fluorescence, Aprepitant at 40 μM for both K562 cells and HL60, was added into the culture medium. Image acquisition continued for 1500 seconds after drug treatment. (C) Quantitative results of Fluo-4 AM and Rhod-2 fluorescence intensity in K562 and HL60 cells in (B). Calcium concentrations were expressed as the average fluorescence intensity of 20 cells/field randomly from at least three fields at each time point. Arrow indicated the time to add Aprepitant. (D) Quantitative results of Fluo-4 AM and Rhod-2 fluorescence intensity in K562 and HL60 cells treated by $H_2O_2$. Calcium concentrations were expressed as the average fluorescence intensity of 20 cells/field randomly from at least three fields at each time point. Arrow indicated the time to add $H_2O_2$.

Supplemental Figure 9

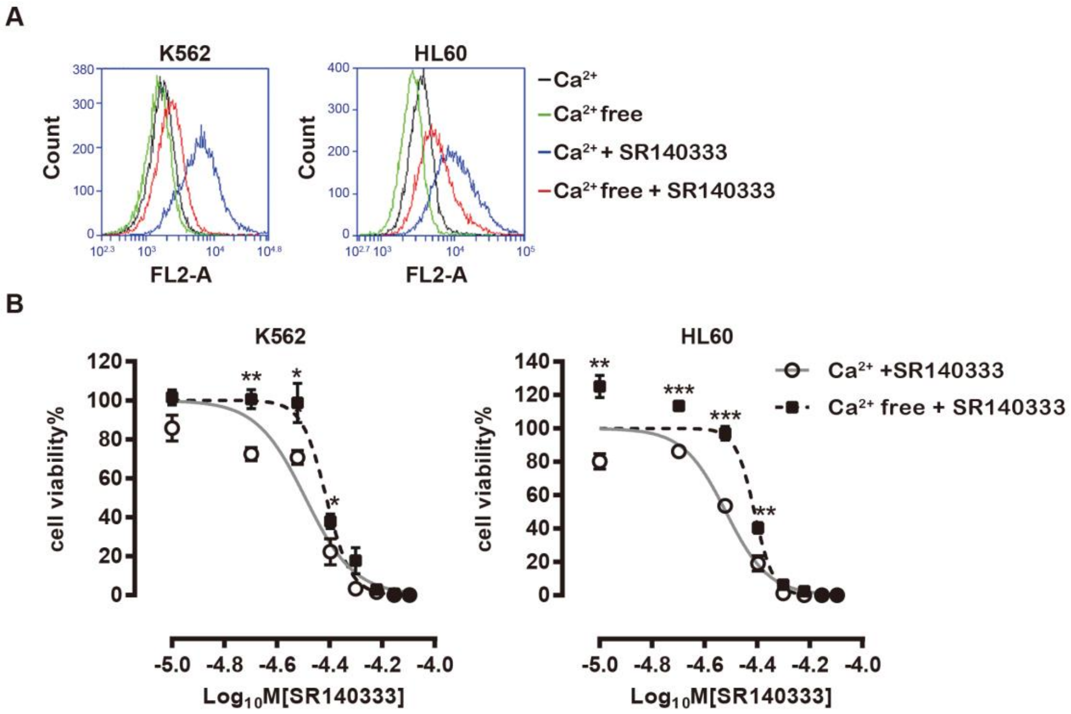


**Figure S9 Mitochondrial calcium flux contributes to oxidative stress and cell death in response to NK-1R inhibition.** (A) K562 and HL60 cells were cultured in the medium in the presence or the absence of $Ca^{2+}$, and then treated with SR140333 at 60 μM and 30 μM, respectively. The mitochondrial superoxide level was measured by MitoSOX three hours after treatment. (B) The cell viability was calculated as the percentage of live cells in the drug treatment group relative to the vehicle group 24 hours after treatment. The live cells were counted by trypan blue exclusion, Values are means ± SEM (n = 3) (C). **P*<0.05, ** *P*<0.01, ****P*<0.001, compared with the group treated with SR140333 in the normal medium.

Supplemental Figure 10

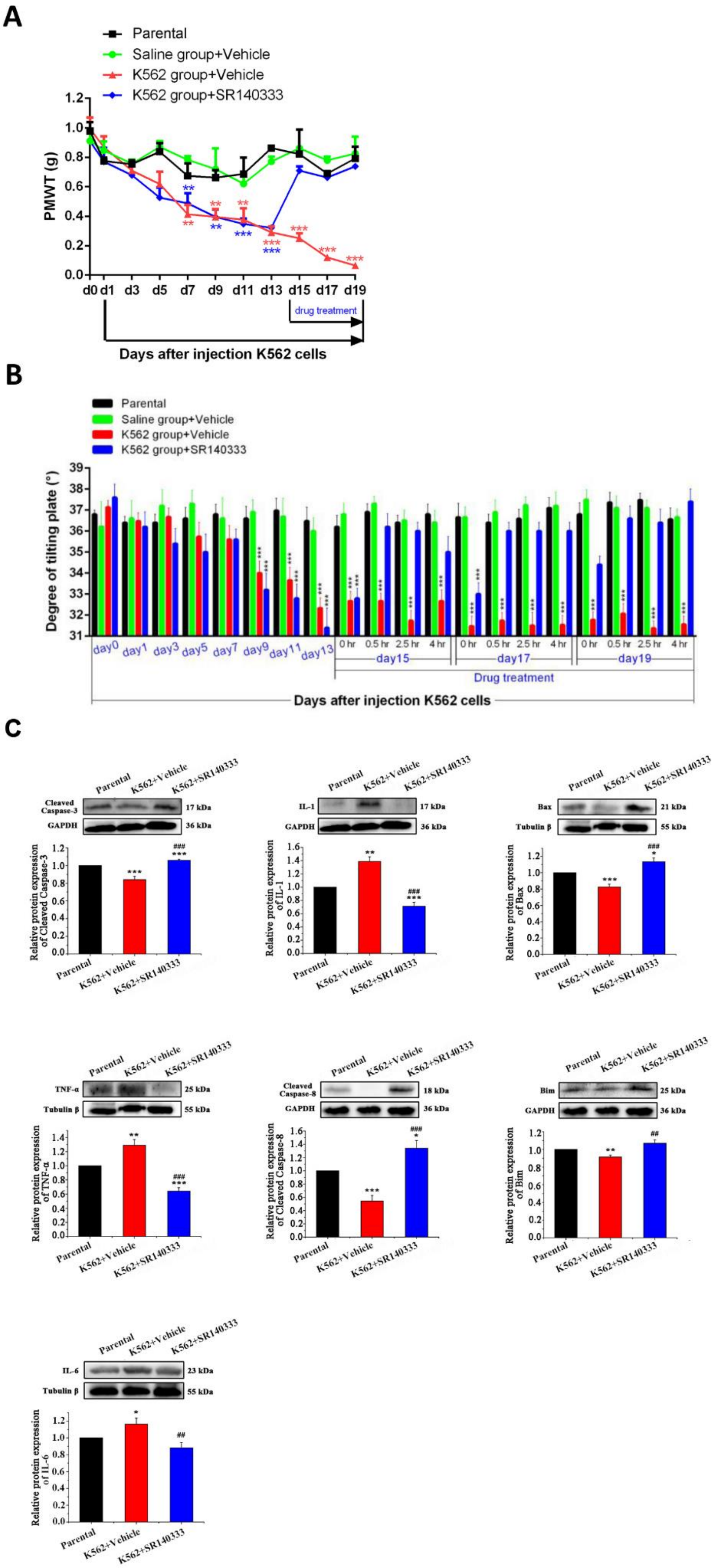

**Figure S10 Blocking NK-1R alleviated leukemia-induced bone pain *in vivo*.** (A) Measurement of 50% hind paw mechanical withdrawal threshold (PMWT) to von Frey filaments. ***P*<0.01, ****P*<0.001, compared with the parental group on the same experiment day. (B) Inclined-plate test to assess muscular strength and the proprioception. Values represent means ±SEM. ** *P*<0.01, ****P*<0.001, compared with the saline group treated with vehicle on the corresponding day. (C) Quantification of apoptosis and inflammation-related proteins by densitometry. GAPDH and Tubulin β were used as the loading controls. For the proteins probed in the same membrane (cleaved caspase-3 and IL-1; Bax and TNF-α; cleaved caspase-8 and Bim), one loading control was used. **P*<0.05, ** *P*<0.01, ****P*<0.001, compared with the parental group; $^{\#\#}$*P*<0.01, $^{\#\#\#}$*P*<0.001, compared with the K562 group treated with vehicle.

**Table S1: The information of myeloid leukemia patients.**

| Patient | Age | Sex | subtype | PB WBC ($\times 10^9$/L) | Scores |
|---|---|---|---|---|---|
| **1** | 70 | male | M2a | 12.7 | 1 |
| **2** | 69 | female | M2a | 4.1 | 2 |
| **3** | 69 | male | M5 | 5.4 | 1 |
| **4** | 66 | female | M2a | 14.5 | 2 |
| **5** | 70 | female | M5 | 97.0 | 3 |
| **6** | 14 | male | M2a | 3.1 | 1 |
| **7** | 26 | female | M5 | 10.3 | 2 |
| **8** | 71 | male | M2a | 3.5 | 2 |
| **9** | 33 | female | M3 | 15.3 | 2 |
| **10** | 26 | male | M2a | 5.3 | 3 |
| **11** | 77 | female | M2a | 19.7 | 1 |
| **12** | 48 | female | M2a | 17.19 | 1 |
| **13** | 29 | female | M2a | 15.65 | 2 |
| **14** | 27 | male | M4EO | 2.57 | 2 |
| **15** | 20 | male | M3 | 15.6 | 3 |
| **16** | 64 | male | M2a | 2.7 | 1 |
| **17** | 67 | female | M3 | 2.1 | 3 |

Note: PB for peripheral blood, WBC for white blood cells.

## References

Chaplan, S., Bach, F., Pogrel, J., Chung, J., and Yaksh, T. (1994). Quantitative assessment of tactile allodynia in the rat paw. Journal of neuroscience methods *53*, 55-63.

Fang, X., Hu, H., Xie, J., Zhu, H., Zhang, D., Mo, W., Zhang, R., and Yu, M. (2012). An involvement of neurokinin-1 receptor in FcεRI-mediated RBL-2H3 mast cell activation. Inflammation Research *61*, 1257-1263.

Fu, C.Y., Zhao, Y.L., Dong, L., Chen, Q., Ni, J.M., and Wang, R. (2008). In vivo characterization of the effects of human hemokinin-1 and human hemokinin-1 (4-11), mammalian tachykinin peptides, on the modulation of pain in mice. Brain, behavior, and immunity *22*, 850-860.

Kim, S.H., and Chung, J.M. (1992). An experimental model for peripheral neuropathy produced by segmental spinal nerve ligation in the rat. Pain *50*, 355-363.

Lu, Y., Zhang, T.F., Shi, Y., Zhou, H.W., Chen, Q., Wei, B.Y., Wang, X., Yang, T.X., Chinn, Y.E., Kang, J., *et al.* (2016). PFR peptide, one of the antimicrobial peptides identified from the derivatives of lactoferrin, induces necrosis in leukemia cells. Sci Rep *6*, 20823.

Ou, S., Zhao, Y.-D., Xiao, Z., Wen, H.-Z., Cui, J., and Ruan, H.-Z. (2011). Effect of lappaconitine on neuropathic pain mediated by P2X 3 receptor in rat dorsal root ganglion. Neurochemistry international *58*, 564-573.

Ren, S., Peng, Z., Mao, J.-H., Yu, Y., Yin, C., Gao, X., Cui, Z., Zhang, J., Yi, K., and Xu, W. (2012). RNA-seq analysis of prostate cancer in the Chinese population identifies recurrent gene fusions, cancer-associated long noncoding RNAs and aberrant alternative splicings. Cell research *22*, 806-821.

Zhu, X.-C., Ge, C.-T., Wang, P., Zhang, J.-L., Yu, Y.-Y., and Fu, C.-Y. (2015). Analgesic effects of lappaconitine in leukemia bone pain in a mouse model. PeerJ *3*, e936.
